# The surprising reaction of (chloro- and methanesulfonyloxy)(phenyl)methylphosphonates and –(phenyl)methylphosphine oxides with potassium diphenylphosphide followed by oxidation

**DOI:** 10.3762/bjoc.22.93

**Published:** 2026-08-17

**Authors:** Zsuzsanna Szalai, Janka Bednárik, András Miskó, Angéla Takács, László Kőhidai, László Drahos, György Keglevich

**Affiliations:** 1 Department of Organic Chemistry and Technology, Faculty of Chemical Technology and Biotechnology, Budapest University of Technology and Economics, Műegyetem rkp. 3., 1111 Budapest, Hungary; 2 Department of Genetics, Cell and Immunobiology, Semmelweis University, Nagyvárad tér 4., 1089 Budapest, Hungary; 3 MS Proteomics Research Group, Research Centre for Natural Sciences, 1117 Budapest, Hungary

**Keywords:** hydroxyphosphonate derivatives, phosphinylation, potassium diphenylphosphide, rearrangement, reversibility

## Abstract

α-Hydroxyphosphonates and α-hydroxyphosphine oxides are important intermediates for additional biologically active species. While (hydroxy(phenyl)methyl)(diphenyl)phosphine oxide did not even undergo chlorination with thionyl chloride, the reaction of diethyl chloro(phenyl)methylphosphonate with potassium diphenylphosphide followed by oxidation with hydrogen peroxide resulted in the expected bis(>P(O)-functionalized) product only as a minor component. The similar reaction of the analogous (methanesulfonyloxy)(phenyl)methyl derivative afforded a mixture of a diethyl (diethoxyphosphonyl)(phenyl)methyl phosphate, a diethyl (diphenylphosphinyl)(phenyl)methyl phosphate, and a ((diphenylphosphinyl)(phenyl)methyl) diphenylphosphinate. The formation of the unexpected products was explained assuming rearrangements and reversible formation of the hydroxymethylene-bis(>P(O)-functionalized) intermediate. Contrary to the earlier experiences, the reaction of ((methanesulfonyloxy)(phenyl)methyl)(diaryl)phosphine oxides with potassium diphenylphosphide followed by oxidation took place in a clear-cut manner providing, with one exception, the corresponding α-phosphinylated α-hydroxyphosphine oxides that could also be synthesized by direct phosphinylation of the starting α-hydroxyphosphine oxide. Cell viability assays performed on U266 myeloma cells revealed concentration-dependent antiproliferative effects of the synthesized compounds on U266 myeloma cells, highlighting the key role of the phosphinoyloxy moiety in the cytotoxic activity, which may be enhanced further by 4-methyl groups in the P-phenyl rings.

## Introduction

α-Hydroxyphosphonates and α-hydroxyphosphine oxides represent a prominent class of compounds due to three reasons [1]. Their synthesis by the Pudovik reaction of oxo compounds and >P(O)H reagents, such as dialkyl phosphites and secondary phosphine oxides still hides challenges for green chemists [1]. Moreover, the adducts obtained in the Pudovik addition may serve as intermediates in a wide range of reactions [2–9]. Last but not least, the α-hydroxyphosphonates and their derivatives may be of real or potential biological activity [10–16]. As regards the reactions of α-hydroxyphosphonates, the Keglevich group found that under microwave (MW) conditions, due to the beneficial effect of the adjacent P=O group, the hydroxy group may be substituted by an amino function in the reaction with primary amines [2–3]. Beyond these unexpected transformations, the hydroxy function was acylated [4], phosphorylated [5–7], and sulfonylated [8]. The sulfonyloxyphosphonates were good starting materials in solvolytic reactions with alcohols to provide the α-alkoxy derivatives [8]. Surprisingly, the sulfonyloxyphosphonates could also be involved in the Michaelis–Arbuzov reaction with trivalent P-esters to furnish bisphosphonic derivatives [8–9]. Hydroxymethylene-bisphosphonates form a special group within α-hydroxyphosphonates, and comprise the corresponding bisphosphonic acids that are called dronic acids, and used in the treatment of bone diseases like osteoporosis [17–20]. We have interest in the synthesis of dronic acid derivatives [21], and also in the preparation of bisphosphonic derivatives in a broader sense to make available newer families and species that may be of biological activity.

For this, in this article, we wish to explore the reaction of α-substituted phosphonates and phosphine oxides with potassium diphenylphosphide. Our hope was to synthesize new bisphosphonic or related derivatives displaying cytotoxic effects.

## Results and Discussion

### Preparative experiments: reaction of α-substituted (phenyl)methylphosphonates and (phenyl)methylphosphine oxides with potassium diphenylphosphide

**Scheme 1:**
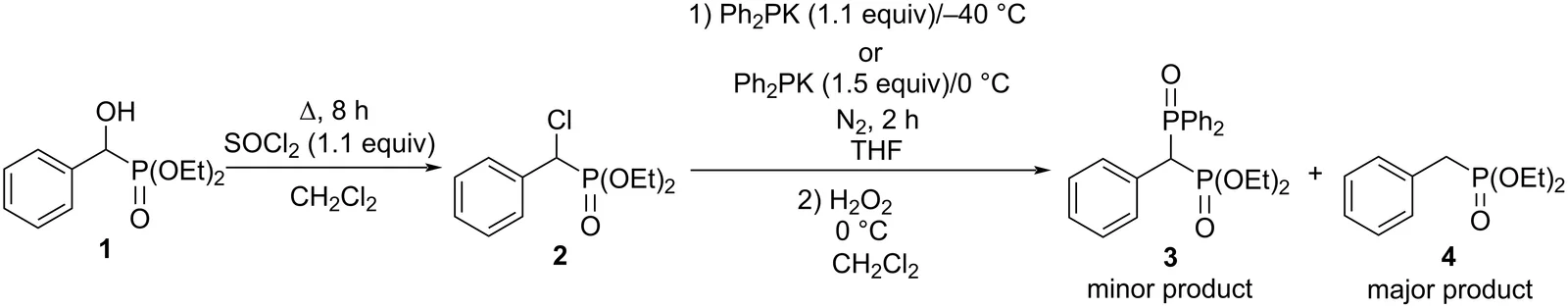
Unexpected outcome of the reaction of diethyl chloro(phenyl)methylphosphonate (**2**) with PDPP followed by oxidation.

**Diethyl chloro(phenyl)methylphosphonate and diethyl (sulfonyloxy)(phenyl)methylphosphonate as starting materials in reactions with potassium diphenylphosphide:** The first attempt was, when diethyl chloro(phenyl)methylphosphonate (**2**), obtained from diethyl hydroxy(phenyl)methylphosphonate (**1**) according to our earlier method [22], was reacted with 1.1 equiv of potassium diphenylphosphide (PDPP) under N_2_ atmosphere in THF. The first run was performed at −40 °C, while the second trial at 0 °C (applying PDPP in the second case in a 1.5 equivalent quantity). The main reaction was then followed by oxidation with 30% hydrogen peroxide. ^31^P NMR and LC–MS analysis of the crude mixtures revealed that at −40 °C, the expected bis(>P(O)-functionalized) product **3** appeared as a minor component (6%) in the crude mixture, while the major component was diethyl phenylmethylphosphonate (**4**: 94%). At 0 °C, diethyl ((diphenylphosphinyl)(phenyl)methyl)phosphonate (**3**) was present in 27%, while phenylmethylphosphonate **4** in 73% (Scheme 1). The latter product (**4**) was formed by dechlorination of diethyl chloro(phenyl)methylphosphonate (**2**). Diphenylphosphine oxide (δ_P_ 21.6, δ_P,lit._ 22.0 [23], [M + H]^+^ = 203), diphenylphosphinic acid (δ_P_ 31.3, δ_P,lit._ 33.8 [24], [M + H]^+^ = 219) and ethyl diphenylphosphinate (δ_P_ 28.4, δ_P,lit._ 31.9 [25], [M + H]^+^ = 247) were also present in the reaction mixtures.

**Scheme 2:**
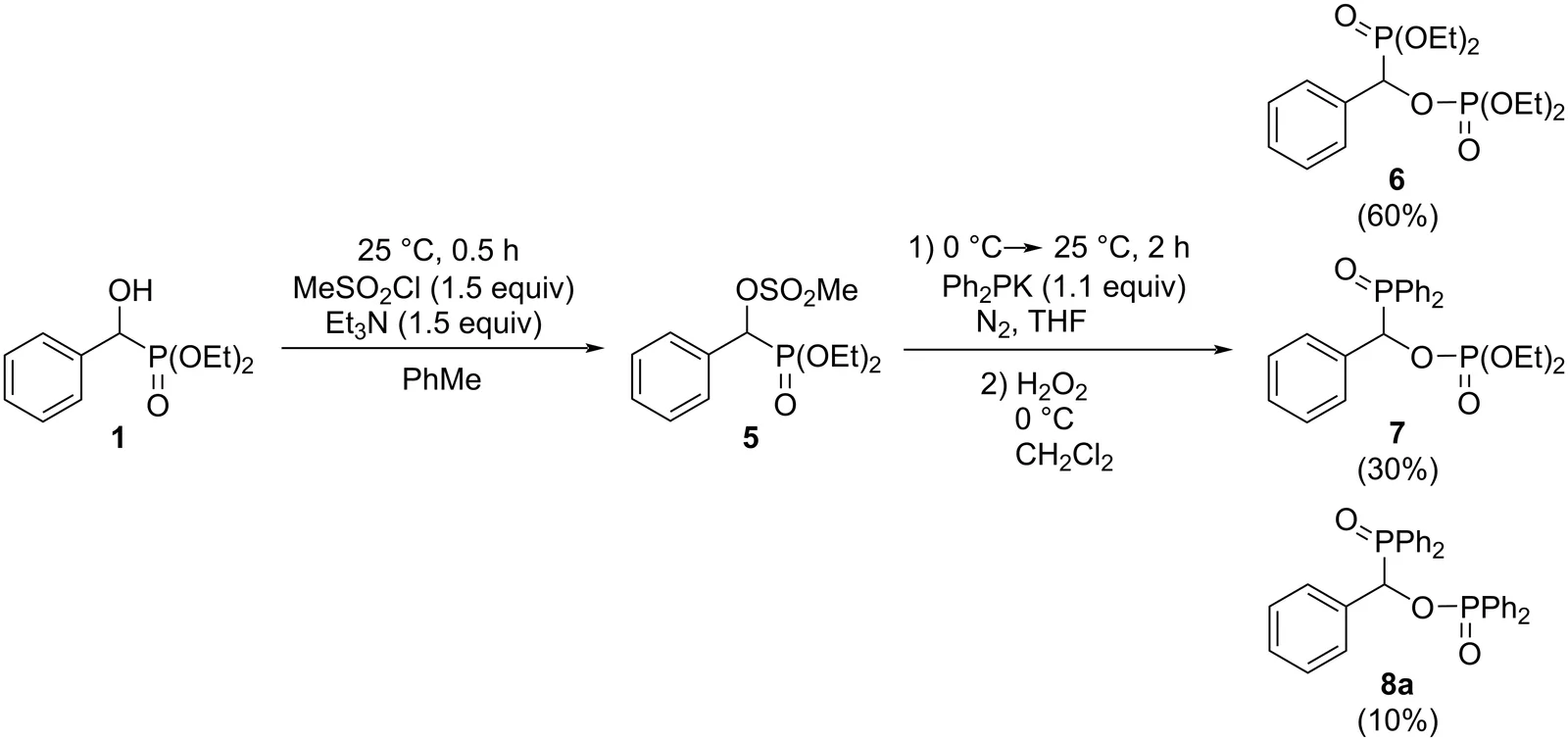
Unexpected outcome of the reaction of (sulfonyloxy)(phenyl)methylphosphonate **5** with PDPP followed by oxidation.

In the second round, (methanesulfonyloxy)(phenyl)methylphosphonate **5** was chosen as the starting material that was prepared by the sulfonylation of hydroxyphosphonate **1** using our method [8]. Then, the sulfonyloxy derivative **5** was reacted with 1.1 equiv of PDPP at 0→25 °C in THF. After oxidation, as above, the crude mixture comprised diethyl (diethoxyphosphonyl)(phenyl)methyl phosphate (**6**, 60%), diethyl (diphenylphosphinyl)(phenyl)methyl phosphate (**7**, 30%), and (diphenylphosphinyl)(phenyl)methyl diphenylphosphinate (**8a**, 10%) (Scheme 2).

**Scheme 3:**
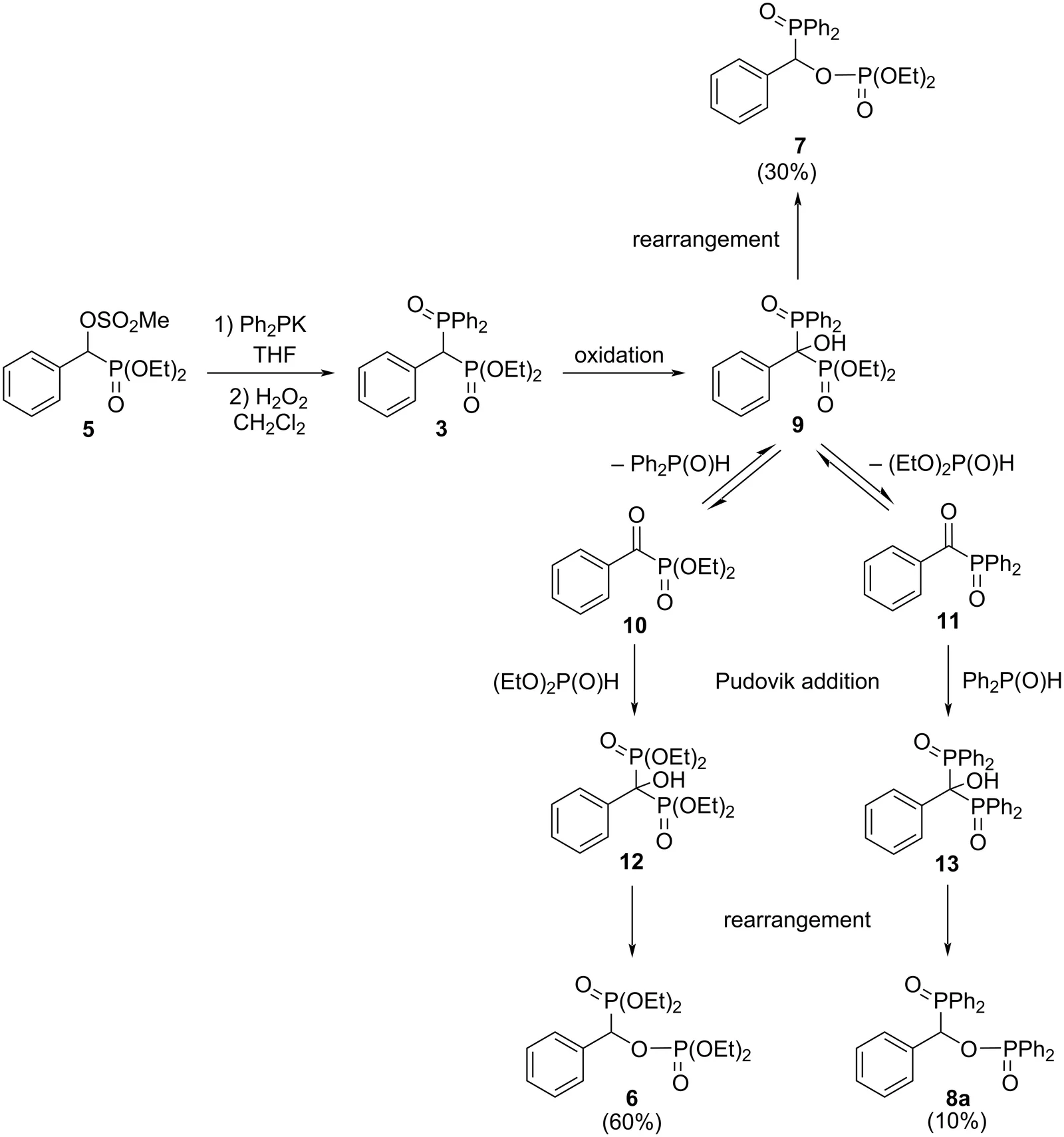
Putative explanation for the formation of compounds **6–8** shown in Scheme 2.

The unexpected outcome of the reaction may be explained as follows. In the first step, the desired substitution may take place, that is followed by the blocking of the trivalent phosphorus atom to pentavalent-tetracoordinated **3**. Then, diethyl ((diphenylphosphinyl)(phenyl)methyl)phosphonate (**3**) undergoes oxidation to furnish bis(>P(O))-functionalized hydroxymethylene derivative **9** that is the key intermediate for further transformations (Scheme 3). A simple rearrangement of species **9** may lead to the minor component, diethyl (diphenylphosphinyl)(phenyl)methyl phosphate (**7**). The rearrangement of hydroxymethylene-bis(>P(O)) species **9** to phosphine oxide–phosphate **7** seems to be selective, as the other possibility, the transfer of the P(O)Ph_2_ moiety to the O atom was not realized. This suggests the dominance of the steric factors over the electronic ones, as although the P atom of the P(O)Ph_2_ unit is more electrophilic than that of the P(O)(OEt)_2_ function, the latter center may be approached easier than the more hindered P(O)Ph_2_ unit. The other possibility is that the bis(>P(O))hydroxymethylene derivative **9**, that is as a matter of fact, the adduct of α-oxophosphonate **10** and diphenylphosphine oxide, or that of α-oxophosphine oxide **11** and diethyl phosphite, may get decomposed to the components mentioned above under the conditions applied. Reversible formation of hydroxy(phenyl)methylphosphonates is a well-known phenomenon [26–27]. Returning to the putative reaction sequence suggested in Scheme 3, the Pudovik reaction of diethyl phosphite and α-oxophosphonate **10**, and the addition of diphenylphosphine oxide on the C=O group of α-oxophosphine oxide **11** provided hydroxymethylenebisphosphonate **12**, and hydroxymethylenebis(phosphine oxide) **13**, respectively, that under the conditions of the reaction undergo rearrangement to give the major product diethyl (diethoxyphosphonyl)(phenyl)methyl phosphate (**6**), and the minor species (diphenylphosphinoyl)(phenyl)methyl diphenylphosphinate (**8a**). The unstability of bis(>P(O)-functionalized hydroxymethylene derivatives, especially with a phenyl group on the central carbon atom was observed by us [28–29]. Traces of product **6**, **7** and **8a** were also detected in the reaction mixture of diethyl chloro(phenyl)methyl)phosphonate (**2**) and PDPP, followed by oxidation (not shown in Scheme 1).

**Scheme 4:**
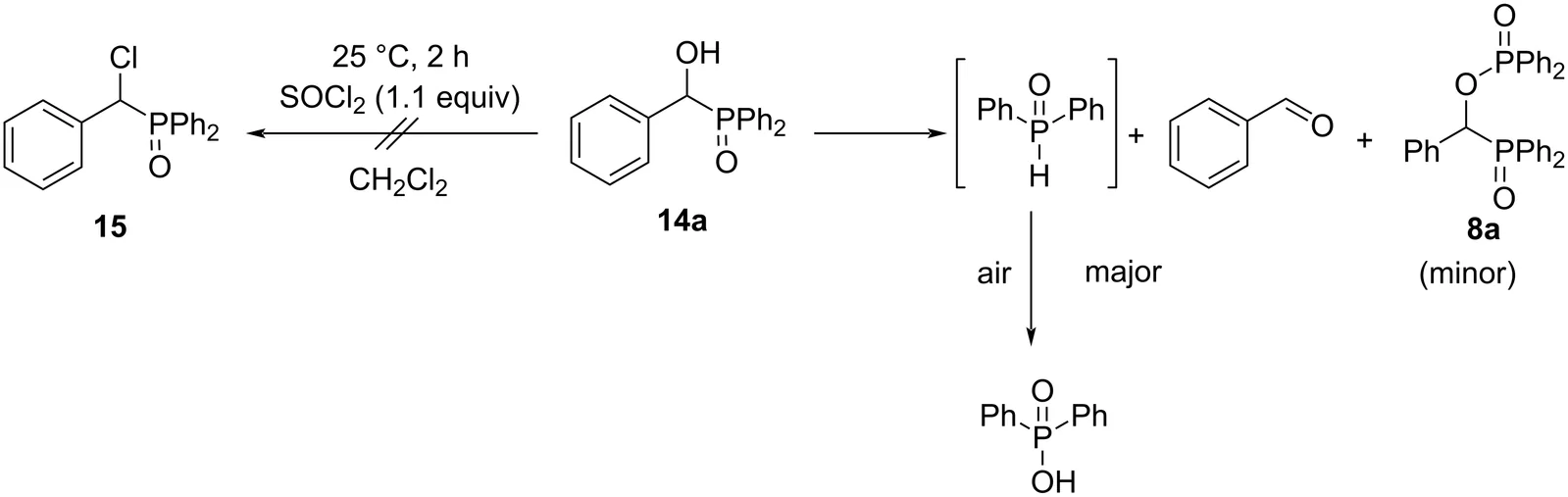
Result of the attempted chloro-substitution of hydroxy(phenyl)methylphosphine oxide **14a**.

**(Chloro(phenyl)methyl)(diphenyl)phosphine oxide and ((sulfonyloxy)(phenyl)methyl)(diaryl)phosphine oxides as starting materials in reaction with potassium diphenylphosphide:** In the next stage of our work, α-substituted (phenyl)methylphosphine oxides were selected as starting materials. However, we failed already at the attempt to convert (hydroxy(phenyl)methyl)(diphenyl)phosphine oxide (**14a**) to the corresponding α-chloro derivative **15** by reaction with thionyl chloride, as under the conditions of the reaction, the starting α-hydroxyphosphine oxide was phosphinoylated to give product **8a** in a 5% proportion, along with diphenylphosphinic acid (δ_P_ 31.3, δ_P,lit._ 33.8 [24], [M + H]^+^ = 219) and benzaldehyde ([M + H]^+^ = 107) as the major components. Ph_2_P(O)OH came from the oxidation of Ph_2_P(O)H under the aerobic conditions (Scheme 4). Benzaldehyde and Ph_2_P(O)H were formed from hydroxyphosphine oxide **14a** as a consequence of the reversibility of the Pudovik reaction discussed above [26–27].

The explanation for the formation of product **8a** may be the following. Under the conditions of the attempted chlorination, a part of the “Pudovik adduct” of benzaldehyde and diphenylphosphine oxide gets decomposed to the components. Reversible formation of the benzaldehyde–diphenylphosphine oxide adduct has been described in [30]. Then, the P-reagent may be chlorinated, and it may react with the remaining hydroxyphosphine oxide **14a** to result in the formation of the phosphinoylated species **8a**. Some oxidation of the secondary phosphine oxide to the diphenylphosphinic acid is inevitable. Chlorination of the latter byproduct may also provide the P-reagent that phosphinoylates the remaining hydroxyphosphine oxide **14a**.

**Scheme 5:**
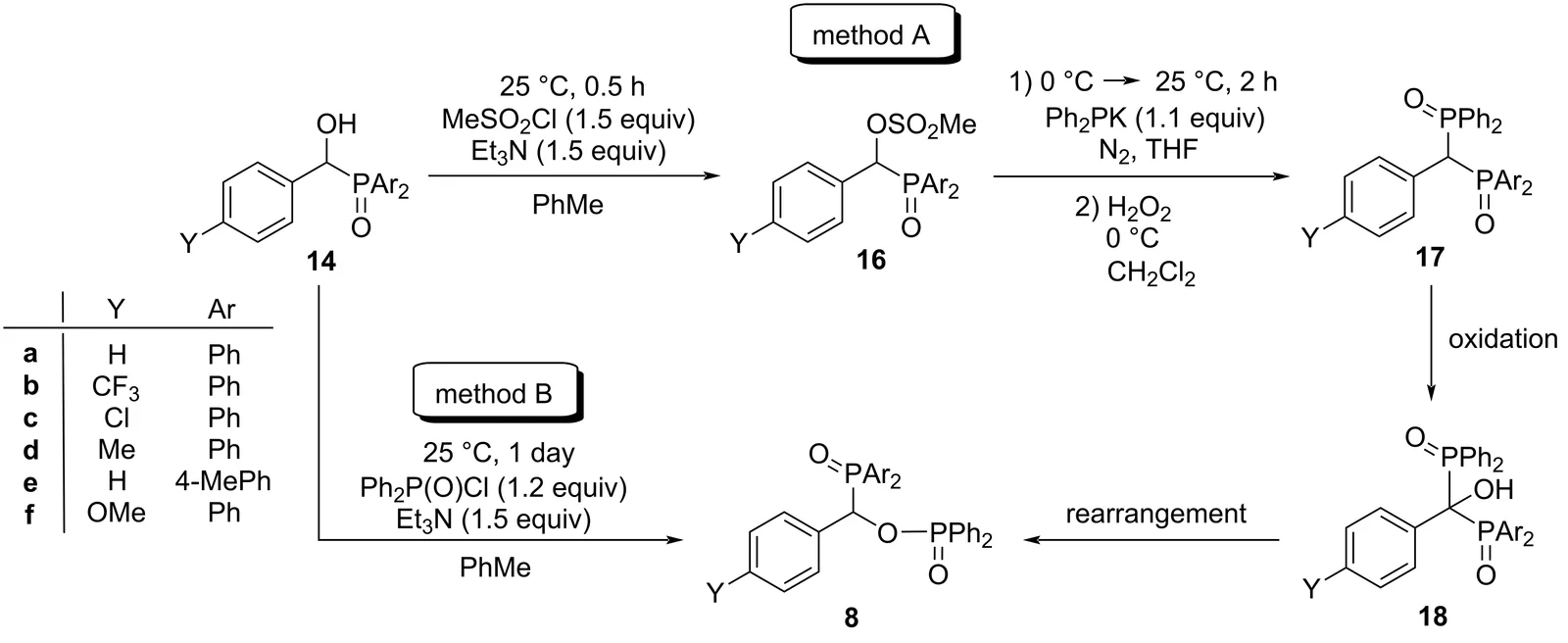
Two pathways (methods A and B) for the preparation of (diarylphosphinyl)(aryl)methyl diphenylphosphinates **8** starting from the corresponding (hydroxy(aryl)methyl)(diaryl)phosphine oxides **14**.

**Scheme 6:**
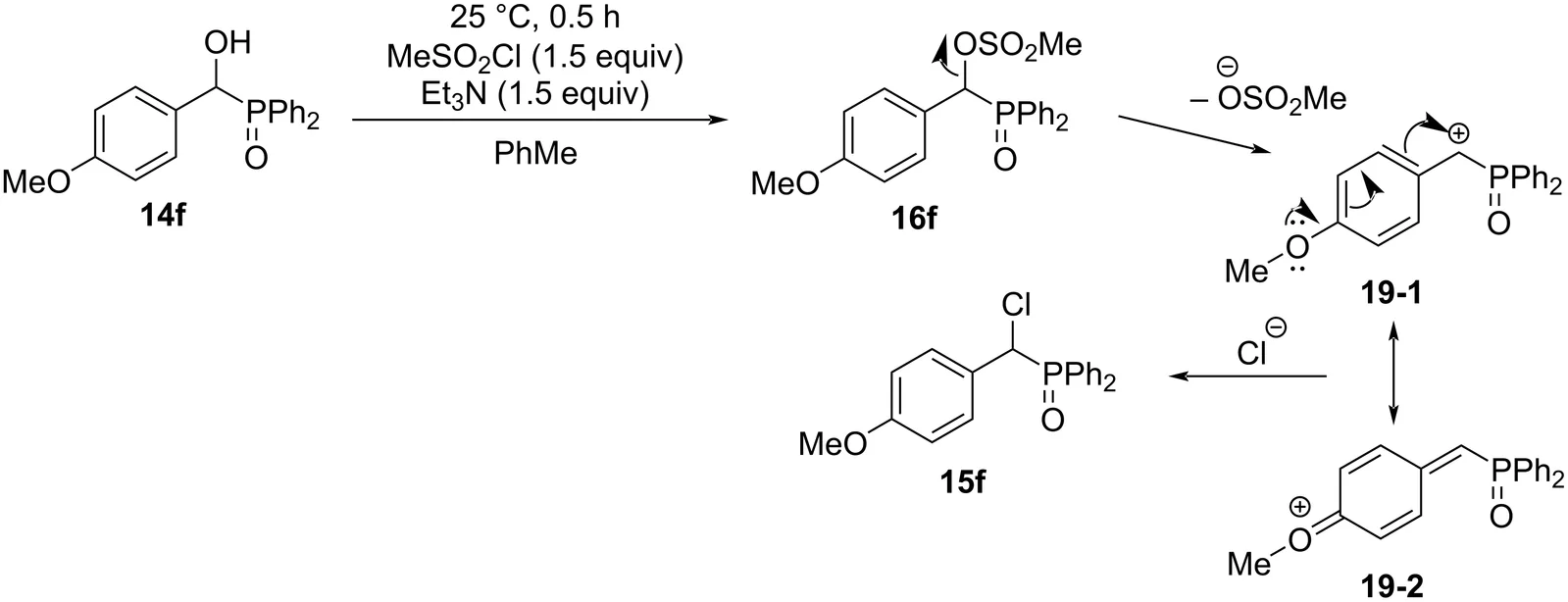
Unexpected route following the mesylation of (hydroxy(4-methoxyphenyl)methyl)(diphenyl)phosphine oxide (**14f**).

Finally, a series of (hydroxy(aryl)methyl)(diaryl)phosphine oxides **14a–e** was prepared by the Pudovik reaction, and were transformed into the methanesulfonylated derivatives **16a–e** by reaction with methanesulfonyl chloride in the presence of triethylamine at 25 °C in toluene (Scheme 5). It was found that the attempted methanesulfonylation of the (hydroxy(4-methoxyphenyl)methyl)phosphine oxide **14f** did not provide the expected product, but the corresponding (chloro(aryl)methyl)phosphine oxide **15f** was formed. This phenomenon has already been observed by us on the analogous example of (hydroxy(4- and 2-methoxyphenyl)methyl)phosphonates [8], and the proposed mechanism involving a quinoide intermediate is shown in Scheme 6.

Interaction of the sulfonyloxy species **16a**–**e** with PDPP at 0→25 °C in THF under N_2_, followed by oxidation with 30% hydrogen peroxide afforded eventually the corresponding (diarylphosphinoyl)(aryl)methyl diphenylphosphinates **8a**–**e**. Hydroxymethylene-bis(>P(O)) species **18** was assumed as an intermediate. Its rearrangement to phosphine oxide–phosphinate **8**, in most cases (when Ar = Ph) may afford a single isomer. However, in the instance, when Ar = 4-MePh, the selectivity may be explained by electronic reasons, as the P atom of the P(O)Ph_2_-function is more electrophilic than that of the P(O)(4-MePh)_2_ moiety. Purification by column chromatography afforded products **8a**–**e** in 37–56% yields. Beside the above approach marked as “method A”, products **8a**–**e** completed with **8f** could also be prepared by the direct phosphinylation of the starting (hydroxy(aryl)methyl)(diaryl)phosphine oxides **14a**–**f** (Scheme 5, “method B”).

The new products (**14b**, **16b**–**d**, **15f**, **8b**–**f**) were fully characterized by ^31^P, ^13^C and ^1^H NMR spectral data, and the elemental composition was confirmed by HRMS.

### Cytotoxic effects of **14a–d**,**f**, **16a**,**e** and **8a**,**c**,**e**,**f** on U266 myeloma cells

**Figure 1:**
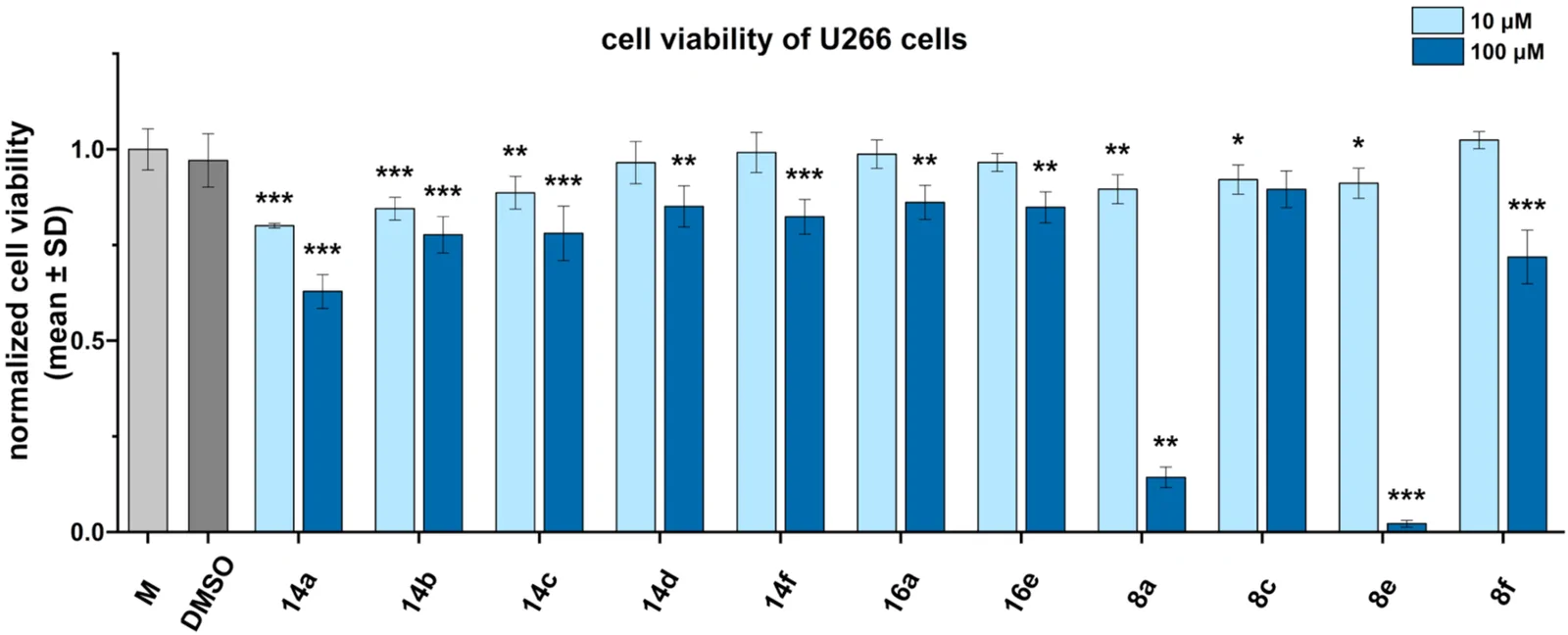
In vitro antiproliferative effects of compounds tested at 10 and 100 µM on U266 cell line after 72 h. The data are normalized to the medium control. Data represented as the mean ± SD; *n* = 3. The levels of significance are shown as follows: *: *p* < 0.05; **: *p* < 0.01; ***: *p* < 0.001, determined by the one-way ANOVA test, followed by Fisher’s LSD post hoc test.

Figure 1 demonstrates that the synthesized compounds induced concentration-dependent decreases in the viability of the U266 myeloma cells after a 72 h treatment. Multiple myeloma is a hematological malignancy characterized by the clonal proliferation of plasma cells, the terminally differentiated effector cells of the B-cell lineage within the bone marrow. Given the established role of phosphorus-containing drugs in the treatment of myeloma-related complications and their potential antitumor effects, this disease represents an attractive target for the development of novel phosphorus-containing therapeutic agents [31]. Moreover, in our previous studies, several phosphonate derivatives synthesized by our group exhibited promising activity against the U266 cell line [9,32].

Consistent with a concentration-dependent response, at 10 µM, most of the compounds caused only moderate reductions in the cell viability, generally maintaining 80–100% survival relative to the untreated control medium. In contrast, treatment of the cell line at 100 µM resulted in more pronounced antiproliferative effects. Several derivatives resulted in statistically significant decreases in the number of viable cells.

(Hydroxy(aryl)methyl)(diaryl)phosphine oxides **14a–d**,**f** and ((methanesulfonyloxy)(aryl)methyl)(diaryl)phosphine oxides **16a** and **16e** induced only moderate reductions, generally maintaining viabilities between ca. 75–90% of the control level at a 100 µM treatment. Among these compounds, derivative **14a** resulted in the strongest effect, reducing the cell viability to 62 ± 4.4%.

Among the tested compounds, the (diphenylphosphinyl)(phenyl)methyl) (bis(4-methylphenyl))phosphinate (**8e**) exhibited the strongest cytotoxic activity, reducing the cell viability to nearly complete loss of survival (characterized by a 2.2 ± 0.8% residual viability) at 100 µM. Compound **8a** also showed a remarkably strong effect, decreasing cell viability to 14.3 ± 2.6% at the higher concentration.

Interestingly, a part of the (diarylphosphinyl)(aryl)methyl diphenylphosphinates **8** proved to be substantially more effective than the starting model compounds, suggesting that replacement of the hydroxy or mesyloxy group with a diphenylphosphinyloxy moiety strongly enhances the cytotoxic activity. Within the series of compound family **8**, the P-diphenyl derivative **8a** and the bis(4-methylphenyl)-substituted **8e** were the most potent. These results indicate that the strong activity does not require substitution at the aryl ring attached to the central carbon, since neither the electron-withdrawing chloro-substituent, nor the electron-donating methoxy group enhanced the potency comapared to the unsubstituted **8a**. However, the presence of the electron-donating 4-methyl group in the phenyl rings attached to the central P-atom, as in compound **8e**, may enhance further the antiproliferative activity.

Overall, the phosphinoylation of the starting (hydroxy(aryl)methyl)(diaryl)phosphine oxides **8** led to a cytotoxic effect on myeloma cells. Hence, the phosphinoyloxy moiety appears to be the primary structural feature associated with the enhanced antiproliferative activity. This effect may be increased further by the introduction of electron-donating methyl-substituents in the *para*-position of the P-phenyl rings. The results suggest that these compounds are suitable candidates for further development and structural refinement.

## Conclusion

The preparation of the ((chloro- and methanesulfonyloxy)(phenyl)methyl)phosphonates, as well as the (chloro- and methanesulfonyloxy)(phenyl)methylphosphine oxides and their subsequent reaction with potassium diphenylphosphide followed by oxidation with hydrogen peroxide revealed a diverse situation. While (hydroxy(phenyl)methyl)(diphenyl)phosphine oxide resisted undergoing chlorination with thionyl chloride, the reaction of diethyl chloro(phenyl)methylphosphonate with potassium diphenylphosphide followed by oxidation resulted in the expected bis(>P(O)-functionalized) product as a minor product. The analogous reaction of the (methanesulfonyloxy)(phenyl)methyl derivative furnished a mixture of three compounds: a diethyl (diethoxyphosphonyl)(phenyl)methyl phosphate, a diethyl (diphenylphosphinyl)(phenyl)methyl phosphate, and a (diphenylphinyl)(phenyl)methyl diphenylphosphinate.

The products could be deduced by assuming rearrangements and reversible formation of the hydroxymethylene-bis(>P(O)-functionalized) intermediate. The reaction sequence of ((methanesulfonyloxy)(phenyl)methyl)(diaryl)phosphine oxides with potassium diphenylphosphide followed by oxidation led to the α-phosphinylated α-hydroxyphosphine oxides that were prepared alternatively by the direct phosphinoylation of the starting α-hydroxyphosphine oxide. Cell viability assays demonstrated that the synthesized compounds exerted concentration-dependent antiproliferative effects on U266 myeloma cells, with derivatives **8e** and **8a** exhibiting the highest activity. These findings suggest that the presence of the phosphinoyloxy moiety is essential for enhanced cytotoxic potency, but 4-methyl substituents in the P-phenyl rings may increase further the antiproliferative effect.

## Supporting Information

File 1 Experimental details and the NMR spectra of the compounds synthesized.

## Data Availability

Data generated and analyzed during this study is available from the corresponding author upon reasonable request.
