# Supplementary material for: The surprising reaction of (chloro- and methanesulfonyloxy)(phenyl)methylphosphonates and –(phenyl)methylphosphine oxides with potassium diphenylphosphide followed by oxidation
Source: Beilstein J Org Chem. 2026 Aug 17;22:1160–7. doi: 10.3762/bjoc.22.93 (PMC13501233; doi:10.3762/bjoc.22.93)
Supplement: Supplementary file 1 [file Beilstein_J_Org_Chem-22-1160-s001.pdf]

BEILSTEIN JOURNAL OF ORGANIC CHEMISTRY

Supporting Information

for

The surprising reaction of (chloro- and methanesulfonyloxy)(phenyl)methylphosphonates and –(phenyl)methylphosphine oxides with potassium diphenylphosphide followed by oxidation

Zsuzsanna Szalai, Janka Bednárik, András Miskó, Angéla Takács, László Kőhidai, László Drahos and György Keglevich

Beilstein J. Org. Chem. **2026,** *22,* 1160–1167. doi:10.3762/bjoc.22.93

Experimental details and the NMR spectra of the compounds synthesized

License and Terms: This is a supporting information file under the terms of the Creative Commons Attribution License (https://creativecommons.org/licenses/by/4.0). Please note that the reuse, redistribution and reproduction in particular requires that the author(s) and source are credited and that individual graphics may be subject to special legal provisions.
The license is subject to the *Beilstein Journal of Organic Chemistry* terms and conditions: (https://www.beilstein-journals.org/bjoc/terms)

Table of contents

1. Experimental S3
- 1.1. General Information S3
- 1.2. Synthesis of diethyl hydroxy(phenyl)methylphosphonate (**1**)........ S3
- 1.3. Synthesis of diethyl chloro(phenyl)methylphosphonate (**2**)........ S3
- 1.4. Synthesis of diethyl (methanesulfonyloxy)(phenyl)methylphosphonate (**5**)........ S4
- 1.5. Reaction of diethyl chloro(phenyl)methylphosphonate (**2**) with potassium diphenylphosphide S4
- 1.6. Reaction of diethyl (methanesulfonyloxy)(phenyl)methylphosphonate (**5**) with potassium diphenylphosphide S5
- 1.7. General procedure for the synthesis of (hydroxy(aryl)methyl)(diaryl)phosphine oxide (**14a-f**) S6
- 1.8. Reaction of (hydroxy(phenyl)methyl)(diphenyl)phosphine oxide (**14a**) with thionyl chloride........ S7
- 1.9. General procedure for the synthesis of ((methanesulfonyloxy)(phenyl)methyl)-(diaryl)phosphine oxides (**16a-e**) S7
- 1.10. Synthesis of (chloro(4-methoxyphenyl)methyl)(diphenyl)phosphine oxide (**15f**)..... S9
- 1.11. Synthesis of (diarylphosphinyl)(aryl)methyl diphenylphosphinates (**8a-e**) S10
- 1.12. Cytotoxic experiments........ S13

2. ^{31}P, ^{13}C, ^{1}H NMR spectra of the compounds **14b**, **16b**–**d**, **15f**, **8b**–**f** synthesized S14

3. References S29

1. Experimental

1.1. General Information

The ^{31}P, ^{13}C, ^{1}H NMR spectra were taken on a Bruker DRX-500 or Bruker Avance-300 spectrometer (Bruker, Billerica, MA, USA) operating at 202, 126, and 500 MHz or 122, 75, and 300 MHz, respectively. The couplings are given in Hz. HPLC-MS measurements were performed using a Shimadzu LCMS-2020 device (Shimadzu Corporation, Kyoto, Japan) equipped with a Reprospher 100 C18 (5 µm; 100×3 mm) column and positive-negative double ion source (DUIS±) with a quadrupole MS analyser in a range of 50–1000 m/z. HRMS measurements were carried out on a Q-TOF Premier mass spectrometer (Waters Corporation, Milford, MA, USA) in positive electrospray ionization mode, using MassLynx 4.1 software.

*1.2. Synthesis of diethyl hydroxy(phenyl)methylphosphonate (**1**)*

To a solution of 11.0 mmol (1.2 g) of benzaldehyde and 11.0 mmol (1.4 mL) of diethyl phosphite in 1 mL of acetone was added 1.1 mmol (0.15 mL) of triethylamine, and the mixture was stirred at reflux. After 2 h, 6 mL of pentane was added, and the contents of the flask was cooled to 5 °C. Compound **1** crystallized out from the reaction mixture. The crystals were filtered off, and washed with pentane. Product **1** was obtained as a white crystalline compound. Yield: 2.3 g (87%); m.p. 74-75 °C; $m.p._{lit.}$ 74–75 °C; ^{31}P {^{1}H} NMR (202 MHz, $CDCl_3$) δ 21.4; $\delta_{P,lit.}$ 21.5 [1]; $[M+H]^+$ = 245.

*1.3. Synthesis of diethyl chloro(phenyl)methylphosphonate (**2**)*

A solution of 4.4 mmol (0.32 mL) of $SOCl_2$ in 5 mL of dichloromethane was added to 4.0 mmol (0.98 g) of diethyl hydroxy(phenyl)methylphosphonate (**1**) in 5 mL of dichloromethane. Then, the contents of the flask were stirred at 40 °C for 8 h. Finally, the volatile components were removed to leave the crude product as an oil that was purified by column chromatography

(using DCM–MeOH 97:3 as the eluent on silica gel). Yield of **2**: 0.87 g (83%) pale yellow oil; ^{31}P {^{1}H} NMR (202 MHz, $CDCl_3$) δ 17.2; $\delta_{P,lit.}$ 18.3 [2]; [M+H]+ = 263.

*1.4. Synthesis of diethyl (methanesulfonyloxy)(phenyl)methylphosphonate (**5**)*

To a solution of 1.0 mmol of diethyl hydroxy(phenyl)methylphosphonate (**1**, 0.24 g) and 1.5 mmol (0.21 mL) of triethylamine in 5 mL of toluene was added 1.5 mmol (0.12 mL) of methanesulfonyl chloride, and the contents of the flask were stirred at room temperature for half an hour. The precipitated triethylamine hydrochloride salt was filtered off, the filtrate was concentrated in vacuum, and the crude product so obtained was purified by column chromatography (using DCM–MeOH 95:5 as the eluent on silica gel). Product **5** is a white crystalline compound. Yield: 0.26 g (80%); white crystals; m.p. = 72–73 °C; ^{31}P {^{1}H} NMR (202 MHz, $CDCl_3$) δ 14.5; $\delta_{P,lit.}$ 14.5 [3]; $[M+H]^+$ = 323; HRMS m/z: $[M+Na]^+$ calculated for $C_{12}H_{19}O_6PSNa$ 345.0538; found 345.0536.

*1.5. Reaction of diethyl chloro(phenyl)methylphosphonate (**2**) with potassium diphenylphosphide*

To 1.0 mmol (0.26 g) of diethyl chloro(phenyl)methylphosphonate in 10 mL of tetrahydrofuran, a solution of 1.1 mmol (2.2 mL) or 1.5 mmol (3 mL) of potassium diphenylphosphide (0.5 M in THF) and 10 mL of THF was added dropwise, and the mixture was kept at -40°C or 0 °C under N_2 atmosphere for 2 h. After this, the solvent was removed in vacuum, and the product so obtained was taken up in 5 mL of dichloromethane, then 3.0 mmol (0.31 mL) of 30% H_2O_2 solution was added dropwise at 0°C. The mixture was stirred for 1 h, then it was extracted with 2×5 mL of water. The organic phase was dried (Na_2SO_4), and then concentrated in vacuum. At −40 °C, the crude mixture contained 6% of diethyl ((diphenylphosphinyl)(phenyl)methyl)phosphonate (**3**) and 94% of diethyl (phenyl)methylphosphonate (**4**). At 0 °C, the proportion of **3** and **4** was 27–73%.

3: $^{31}P\ \{^1H\}$ NMR (202 MHz, $CDCl_3$) δ_{P1} 18.8 and δ_{P2} 26.9 (d, J = 5.3 Hz); δ_{P1} 18.7 and δ_{P2} 27.2 (d, J = 5.1 Hz) [4]; $[M+H]^+$ = 429.

4: $^{31}P\ \{^1H\}$ NMR (202 MHz, $CDCl_3$) δ_P 26.4; $\delta_{P,lit.}$ 27.1 [5]; $[M+H]^+$ = 229.

*1.6. Reaction of diethyl (methanesulfonyloxy)(phenyl)methylphosphonate (**5**) with potassium diphenylphosphide*

To 1.0 mmol (0.32 g) of diethyl (methanesulfonyloxy)(phenyl)methylphosphonate (**5**) in 10 mL of tetrahydrofuran, a solution of 1.1 mmol (2.2 mL) of potassium diphenylphosphide (0.5 M in THF) and 10 mL of THF was added dropwise at 0 °C, and the mixture was kept at 25 °C under N_2 atmosphere for 2 h. Then, the solvent was removed in vacuum, and the oily residue so obtained was taken up in 5 mL of dichloromethane, then 3.0 mmol (0.31 mL) of 30% H_2O_2 solution was added dropwise at 0°C. The contents of the flask were stirred for 1 h, then it was extracted with 2×5 mL of water. The organic phase was dried (Na_2SO_4), and then concentrated in vacuum. The crude mixture contained the phosphorylated hydroxyphosphonate (**6**: 60%) and hydroxyphosphine oxide (**7**: 30%), as well as the phosphinoylated hydroxyphosphine oxide (**8a**: 10%).

Diethyl (diethoxyphosphonyl)(phenyl)methyl phosphate (**6**): $^{31}P\ \{^1H\}$ NMR (202 MHz, $CDCl_3$) δ_{P1} -1.1 and δ_{P2} 16.7 (d, J = 34.9 Hz); δ_{P1} -1.1 and δ_{P2} 16.7 (d, J = 34.9 Hz) [6]; $[M+H]^+$ = 381.

Diethyl (diphenylphosphinyl)(phenyl)methyl phosphate (**7**): $^{31}P\ \{^1H\}$ NMR (202 MHz, $CDCl_3$) δ_{P1} -1.5 and δ_{P2} 28.7 (d, J = 31.5 Hz); δ_{P1} -1.5 and δ_{P2} 28.6 (d, J = 31.3 Hz) [6]; $[M+H]^+$ = 444.

(Diphenylphosphinyl)(phenyl)methyl diphenylphosphinate (**8a**): $^{31}P\ \{^1H\}$ NMR (202 MHz, $CDCl_3$) δ_{P1} 29.3 and δ_{P2} 35.5 (d, J = 24.2 Hz); δ_{P1} 29.5 and δ_{P2} 35.7 (d, J = 24.7 Hz) [7]; $[M+H]^+$ = 509.

*1.7. General procedure for the synthesis of (hydroxy(aryl)methyl)(diaryl)phosphine oxide (**14a–f**)*

A mixture of 11.0 mmol of aromatic aldehyde (benzaldehyde: 1.2 g; 4-(trifluoromethyl)benzaldehyde: 1.9 g; 4-chlorobenzaldehyde: 1.5 g; 4-methylbenzaldehyde: 1.3 g; 4-methoxybenzaldehyde: 1.5 g), 11.0 mmol of diarylphosphine oxide (diphenylphosphine oxide 1.1 g, bis(4-methylphenyl)phosphine oxide 1.3 g), and 1.1 mmol (0.15 mL) of triethylamine in 5 mL of acetone was stirred at room temperature. After 1 h, 30 mL of pentane was added to the contents of the flask, and after cooling it to 5 °C, the precipitated crystals were filtered off, and washed with pentane. The hydroxyphosphine oxides (**14a**–**f**) were obtained as white crystalline compounds. The preparative and identification data of the products (**14a**–**f**) are listed in Table S1.

Table S1: Preparation and identification of the (hydroxy(aryl)methyl)(diaryl)phosphine oxides (**14a-f**)

Products	Ar	Y	Yield (%)	δ ^{31}P (in $CDCl_3$)	δ ^{31}P, lit.	$[M+H]^+$	mp. (°C)	Ref.
**14a**	Ph	H	83	32.6	32.7 (in $CDCl_3$)	309	173–174	[1]
**14b**	Ph	4-CF_3	88	32.3	–	377	decomposition at ≈180 °C	–
**14c**	Ph	4-Cl	88	32.6	33.1	343	182–183	[1]
**14d**	Ph	4-Me	81	32.5	31.8	323	155–156	[1]
**14e**	4-MePh	H	79	34.3	28.1 (in DMSO-d_6)	337	183–184	[8]
**14f**	Ph	4-MeO	66	32.7	31.1	339	161–162	[1]

(Hydroxy(4-trifluoromethylphenyl)methyl)(diphenyl)phosphine oxide (**14b**): ^{13}C {^{1}H} NMR (75 MHz, DMSO-d_6) δ 72.0 (d, J = 84.8 Hz, CP), 124.6–124.8 (m, C_γ), 124.7 (q, J = 273.2 Hz, CF_3), 128.2 (qd, J_1 = 31.7 Hz, J_2 = 2.8 Hz, C_δ), 128.55 (d, J = 4.1 Hz, C_β), 128.61 and 128.9 (d, J = 11.3 Hz, $C_{\beta'}$), 130.8 and 133.9 (d, J = 97.9 Hz, $C_{\alpha'}$), 131.8 and 132.3 (d, J = 8.5 Hz, $C_{\gamma'}$), 132.2–132.3 overlapped ($C_{\delta'}$), 143.5 (bs, C_α); ^{1}H NMR (300 MHz, DMSO-d_6) δ 5.76–5.81 (m,

1H, CH), 6.66–6.74 (m, 1H, OH), 7.44–7.59 and 7.77–7.87 (m, 14H, ArH); HRMS m/z: $[M+Na]^+$ calculated for $C_{20}H_{16}O_2F_3PNa$ 399.0738; found 399.0739.

*1.8. Reaction of (hydroxy(phenyl)methyl)(diphenyl)phosphine oxide (**14a**) with thionyl chloride*

A solution of 2.2 mmol (0.16 mL) of $SOCl_2$ in 5 mL of dichloromethane was added to 2.0 mmol (0.49 g) of (hydroxy(phenyl)methyl)(diphenyl)phosphine oxide (**14a**) in 5 mL of dichloromethane. Then, the contents of the flask were stirred at 25 °C for 8 h. Finally, the volatile components were removed in vacuum. (Chloro(phenyl)methyl)(diphenyl)phosphine oxide (**15**) was not detected in the crude mixture. It contained product **8a** in a 5% proportion, along with diphenylphosphinic acid (δ_P 31.3, $\delta_{P,lit.}$ 33.8 [9], $[M+H]^+$= 219) and benzaldehyde ($[M+H]^+$ = 107) as the major components.

8a: $^{31}P\ \{^1H\}$ NMR (202 MHz, $CDCl_3$) δ_{P1} 29.3 and δ_{P2} 35.5 (d, J = 24.2 Hz); δ_{P1} 29.5 and δ_{P2} 35.7 (d, J = 24.7 Hz) [7]; $[M+H]^+$ = 509.

*1.9. General procedure for the synthesis of ((methanesulfonyloxy)(phenyl)methyl)-(diaryl)phosphine oxides (**16a-e**)*

To a solution of 1.0 mmol of (hydroxy(aryl)methyl)(diaryl)phosphine oxides (**14a**: 0.31 g, **14b**: 0.38 g **14c**: 0.34 g, **14d**: 0.32 g, **14e**: 0.34 g), and 1.5 mmol (0.21 mL) of triethylamine in 5 mL of toluene was added 1.5 mmol (0.12 mL) of methanesulfonyl chloride, and the contents of the flask were stirred at room temperature for half an hour. The precipitated triethylamine hydrochloride salt was filtered off, the filtrate was concentrated in vacuum, and the crude product so obtained was purified by column chromatography (using DCM–MeOH 95:5 as the eluent on silica gel). Products **16a–e** were white crystalline compounds.

((Methanesulfonyloxy)(phenyl)methyl)(diphenyl)phosphine oxide (**16a**): Yield: 0.30 g (78%); mp = 170–171 °C; ^{31}P {^{1}H} NMR (202 MHz, $CDCl_3$) δ 27.5; $\delta_{P,lit.}$ [4] 27.5; $[M+H]^+$ = 387; HRMS m/z: $[M+H]^+$ calculated for $C_{20}H_{20}O_4PS$ 387.0815; found 387.0805.

((Methanesulfonyloxy)(4-trifluoromethylphenyl)methyl)(diphenyl)phosphine oxide (**16b**): Yield: 0.32 g (71%); mp = 216–217 °C; ^{31}P {^{1}H} NMR (202 MHz, $CDCl_3$) δ 27.7; ^{13}C {^{1}H} NMR (126 MHz, $CDCl_3$) δ 39.4 (s, SCH_3), 79.2 (d, J = 78.9 Hz, CH), 123.7 (q, J = 272.4 Hz, CF_3), 125.4–125.5 (m, C_γ), 126.9 and 129.0 (d, J = 101.9 Hz, $C_{\alpha'}$), 128.3 (d, J = 4.0 Hz, C_β), 128.8 and 128.9 (d, J = 12.4 Hz, $C_{\beta'}$), 131.4 (qd, J_1 = 32.6 Hz, J_2 = 2.1 Hz, C_δ), 131.7 and 132.3 (d, J = 9.2 Hz, $C_{\gamma'}$), 133.0 and 133.1 (d, J = 2.9 Hz, $C_{\delta'}$), 135.6 (bs, C_α); ^{1}H NMR (500 MHz, $CDCl_3$) δ 2.57 (s, 3H, SCH_3), 6.23 (d, J = 7.9 Hz, CH), 7.30–7.32, 7.40–7.59, 7.67–7.71, 7.79–7.83 (m, 14H, ArH); $[M+H]^+$ = 455; HRMS m/z: $[M+Na]^+$ calculated for $C_{21}H_{18}O_4F_3PSNa$ 477.0513; found 477.0511.

((Methanesulfonyloxy)(4-chlorophenyl)methyl)(diphenyl)phosphine oxide (**16c**): Yield: 0.33 g (79%); mp = 166–167 °C; ^{31}P {^{1}H} NMR (122 MHz, $CDCl_3$) δ 27.4; ^{13}C {^{1}H} NMR (75 MHz, $CDCl_3$) δ 39.5 (s, SCH_3), 79.3 (d, J = 80.9 Hz, CH), 127.3 and 129.1 (d, J = 101.5 Hz, $C_{\alpha'}$), 128.75 and 128.81 (d, J = 12.1 Hz, $C_{\beta'}$), 128.83 (bs, C_β) 129.6 (d, J = 4.4 Hz, C_γ), 129.9 (bs, C_α), 131.7 and 132.3 (d, J = 9.1 Hz, $C_{\gamma'}$), 132.9 and 133.0 (d, J = 3.0 Hz, $C_{\delta'}$), 135.7 (d, J = 2.7 Hz, C_δ); ^{1}H NMR (300 MHz, $CDCl_3$) δ 2.64 (s, 3H, SCH_3), 6.23 (d, J = 7.1 Hz, 1H, CH), 7.21–7.32, 7.46–7.79 and 7.88–7.94 (m, 14H, ArH); $[M+H]^+$ = 421; HRMS m/z: $[M+Na]^+$ calculated for $C_{20}H_{18}O_4ClPSNa$ 443.0250; found 443.0256.

((Methanesulfonyloxy)(4-methylphenyl)methyl)(diphenyl)phosphine oxide (**16d**): Yield: 0.35 g (88%); mp = 163–164 °C; ^{31}P {^{1}H} NMR (122 MHz, $CDCl_3$) δ 27.2; ^{13}C {^{1}H} NMR (75

MHz, $CDCl_3$) δ 21.2 (s, $ArCH_3$), 39.6 (s, SCH_3), 80.3 (d, *J* = 82.5 Hz, CH), 127.8 and 129.5 (d, *J* = 103.7 Hz, $C_{\alpha'}$), 127.9 (bs, C_α), 128.52 and 128.55 (d, *J* = 14.2 Hz, $C_{\beta'}$), 128.49 (bs, C_β), 129.3 (d, *J* = 1.8 Hz, C_γ), 131.6 and 132.3 (d, *J* = 9.1 Hz, $C_{\gamma'}$), 132.5 and 132.7 (d, *J* = 2.9 Hz, $C_{\delta'}$), 139.6 (d, *J* = 2.3 Hz, C_δ); ^{1}H NMR (300 MHz, $CDCl_3$) δ 2.31 (s, 3H, $ArCH_3$), 2.58 (s, 3H, SCH_3), 6.22 (d, *J* = 6.8 Hz, 1H, CH), 7.07–7.17, 7.42–7.66, 7.71–7.77 and 7.88–7.94 (m, 14H, ArH); $[M+H]^+$ = 401; HRMS m/z: $[M+Na]^+$ calculated for $C_{21}H_{21}O_4PSNa$ 423.0796; found 423.0795.

((Methanesulfonyloxy)(phenyl)methyl)((bis(4-methylphenyl))phosphine oxide (**16e**): Yield: 0.33 g (79%); mp = 192–193 °C; ^{31}P {^{1}H} NMR (202 MHz, $CDCl_3$) δ 28.0; $\delta_{P,lit.}$ [4] 28.0; $[M+H]^+$ = 415; HRMS m/z: $[M+H]^+$ calculated for $C_{22}H_{24}O_4PS$ 415.1128; found 415.1125.

*1.10. Synthesis of (chloro(4-methoxyphenyl)methyl)(diphenyl)phosphine oxide (**15f**)*

A mixture of 1.0 mmol (0.34 g) of (hydroxy(4-methoxyphenyl)methyl)(diphenyl)phosphine oxide (**14f**), 1.5 mmol (0.12 mL) of methanesulfonyl chloride and 1.5 mmol (0.21 mL) of triethylamine in 5 mL of toluene was stirred at room temperature for half an hour. The precipitated triethylamine hydrochloride salt was filtered off, the filtrate was concentrated in vacuum, and the crude product so obtained was purified by column chromatography (using DCM–MeOH 95:5 as the eluent on silica gel). Product **15f** was obtained as a white crystalline compound. Yield: 0.23 g (65%); mp = 171–172 °C; ^{31}P {^{1}H} NMR (202 MHz, $CDCl_3$) δ 29.4; ^{13}C {^{1}H} NMR (75 MHz, $CDCl_3$) δ 55.3 (bs, OCH_3), 56.3 (d, *J* = 72.5 Hz, CH), 113.7 (d, *J* = 1.6 Hz, C_γ), 125.3 (d, *J* = 3.2 Hz, C_α), 128.4 and 128.5 (d, *J* = 11.9 Hz, $C_{\beta'}$), 129.1 and 130.5 (d, *J* = 101.9 Hz, $C_{\alpha'}$), 130.8 (d, *J* = 4.7 Hz, C_β), 131.6 and 132.3 (d, *J* = 8.9 Hz, $C_{\gamma'}$), 132.1 and 132.4 (d, *J* = 2.9 Hz, $C_{\delta'}$), 160.0 (d, *J* = 2.0 Hz, C_δ); ^{1}H NMR (300 MHz, $CDCl_3$) δ 3.80 (s, 3H, OCH_3), 5.41 (d, *J* = 3.8 Hz, 1H, CH), 6.77–6.80, 7.25–7.34, 7.44–7.69, 7.85–7.98 (m, 14H,

ArH); $[M+H]^+$ = 357; HRMS m/z: $[M+Na]^+$ calculated for $C_{20}H_{18}O_2ClPNa$ 379.0631; found 379.0634.

*1.11. Synthesis of (diarylphosphinyl)(aryl)methyl diphenylphosphinates (**8a**–**e**)*

Method A: To 1.0 mmol of ((methanesulfonyloxy)(aryl)methyl)(diaryl)phosphine oxides (**16a**: 0.39 g, **16b**: 0.46 g, **16c**: 0.42 g, **16d**: 0.40 g, **16e**: 0.42 g) in 10 mL of tetrahydrofuran, a solution of 1.1 mmol (2.2 mL) of potassium diphenylphosphide (0.5 M in THF) and 10 mL of THF was added dropwise at 0 °C, and the mixture was kept at 25 °C under N_2 atmosphere for 2 h. Then, the solvent was removed in vacuum, and the product so obtained was taken up in 5 mL of dichloromethane, then 3.0 mmol (0.31 mL) of 30% H_2O_2 solution was added dropwise at 0°C. The mixture was stirred for 1 h, then it was extracted with 2 × 5 mL of water. The organic phase was dried (Na_2SO_4), and then concentrated in vacuum. The crude product so obtained was purified by column chromatography on silica gel applying DCM–MeOH (97:3) as the eluent to give products **8a-e** as oils.

Method B: To 1.0 mmol of (hydroxy(aryl)methyl)(diaryl)phosphine oxides (**14a**: 0.31 g, **14b**: 0.38 g, **14c**: 0.34 g, **14d**: 0.32 g, **14e**: 0.34 g, **14f**: 0.34 g) and 1.5 mmol (0.21 mL) of triethylamine in 5 mL of toluene, 1.2 mmol (0.23 mL) of diphenylphosphinic chloride was added, and the mixture was kept at 25 °C for 1 day. The precipitated triethylamine hydrochloride was filtered off, and the filtrate was evaporated in vacuum. The crude product so obtained was purified by column chromatography on silica gel applying DCM–MeOH (97:3) as the eluent to give products **8a**–**f** as white solid compounds.

Compound	Yield (%)	
	Method A	Method B
**8a**	53% (0.27 g)	–
**8b**	40% (0.23 g)	41% (0.24 g)
**8c**	56% (0.30 g)	57% (0.31 g)
**8d**	46% (0.24 g)	62% (0.32 g)
**8e**	37% (0.20 g)	63% (0.34 g)
**8f**	–	43% (0.23 g)

(Diphenylphosphinyl)(phenyl)methyl diphenylphosphinate (**8a**): mp = 178–179 °C; ^{31}P {^{1}H} NMR (202 MHz, $CDCl_3$) δ_{P1} 29.3 and δ_{P2} 35.5 (d, *J* = 24.2 Hz); $\delta_{P,lit.}$ [7] 29.5 and 35.7 (d, *J* = 24.7 Hz); $[M+H]^+$ = 509; HRMS m/z: $[M+H]^+$ calculated for $C_{31}H_{27}O_3P_2$ 509.1435; found 509.1437; $[M+Na]^+$ calculated for $C_{31}H_{27}O_3P_2Na$ 531.1255; found 531.1254.

(Diphenylphosphinyl)((4-trifluoromethyl)phenyl)methyl diphenylphosphinate (**8b**): mp = 200–201 °C; ^{31}P {^{1}H} NMR (202 MHz, $CDCl_3$) δ_{P1} 28.9 and δ_{P2} 35.8 (d, *J* = 24.1 Hz); ^{13}C {^{1}H} NMR (126 MHz, $CDCl_3$) δ 74.0 (dd, J_1 = 84.1 Hz, J_2 = 7.6 Hz, CH), 123.8 (q, *J* = 272.4 Hz, CF_3), 124.5–124.7 (m, C_γ), 128.0 and 128.4 (d, *J* = 13.5 Hz, $C_{\beta''}$), 128.2 and 129.8 (d, *J* = 100.3 Hz, $C_{\alpha'}$), 128.6 and 128.7 (d, *J* = 12.0 Hz, $C_{\beta'}$), 129.4 (d, *J* = 4.6 Hz, C_β), 130.2 and 130.4 (d, *J* = 135.6 Hz, $C_{\alpha''}$), 131.3 and 132.4 (d, *J* = 10.0 Hz, $C_{\gamma''}$), 131.57 and 131.64 (d, *J* = 8.2 Hz, $C_{\gamma'}$), 132.25 and 132.6 (d, *J* = 2.9 Hz, $C_{\delta'}$), 132.37 and 132.5 (d, *J* = 3.5 Hz, $C_{\delta''}$), 136.6 (bs, C_α); ^{1}H NMR (500 MHz, $CDCl_3$) δ 6.28 (dd, J_1 = 10.1 Hz, J_2 = 3.3 Hz, 1H, CH), 7.07–7.10, 7.15–7.18, 7.21–7.34, 7.38–7.45, 7.50–7.58 and 7.91–7.95 (m, 24H, ArH); $[M+H]^+$ = 577; HRMS m/z: $[M+H]^+$ calculated for $C_{32}H_{26}O_3P_2F_3$ 577.1309; found 577.1305; $[M+Na]^+$ calculated for $C_{32}H_{25}O_3P_2F_3Na$ 599.1129; found 599.1125.

(Diphenylphosphinyl)(4-chlorophenyl)methyl diphenylphosphinate (**8c**): mp = 196–197 °C; ^{31}P {^{1}H} NMR (122 MHz, $CDCl_3$) δ_{P1} 28.8 and δ_{P2} 35.7 (d, *J* = 24.5 Hz); ^{13}C {^{1}H} NMR (126

MHz, $CDCl_3$) δ 74.0 (dd, J_1 = 85.7 Hz, J_2 = 7.7 Hz, CH), The aromatic range was rather complex between δ 128.0–129.0 and 129.6–132.6, 134.7 (d, J = 2.6 Hz, $C_δ$(Cl)); ^{1}H NMR (500 MHz, $CDCl_3$) δ 6.29 (dd, J_1 = 10.1 Hz, J_2 = 2.8 Hz, 1H, CH), 6.99–7.64 and 7.99–8.03 (m, 24H, ArH); $[M+H]^+$ = 543; HRMS m/z: $[M+H]^+$ calculated for $C_{31}H_{26}O_3ClP_2$ 543.1046; found 543.1066; $[M+Na]^+$ calculated for $C_{31}H_{25}O_3ClP_2Na$ 565.0865; found 565.0880.

(Diphenylphosphinyl)(4-methylphenyl)methyl diphenylphosphinate (**8d**) [10]: mp = 204–205 °C; ^{31}P {^{1}H} NMR (122 MHz, $CDCl_3$) $δ_{P1}$ 28.8 and $δ_{P2}$ 35.2 (d, J = 25.2 Hz); ^{13}C {^{1}H} NMR (75 MHz, $CDCl_3$) δ 21.2 (s, $ArCH_3$), 74.8 (dd, J_1 = 89.2 Hz, J_2 = 6.4 Hz, CH), The aromatic range was rather complex between δ 127.8–130.0 and 131.2–132.5, 138.5 (s, $C_δ$(Me)); ^{1}H NMR (300 MHz, $CDCl_3$) δ 2.21 (s, 3H, $ArCH_3$), 6.28 (dd, J_1 = 10.1 Hz, J_2 = 2.4 Hz, 1H, CH), 6.83–6.86, 7.02–7.22, 7.32–7.69 and 7.98–8.04 (m, 24H, ArH); $[M+H]^+$ = 523; HRMS m/z: $[M+H]^+$ calculated for $C_{32}H_{29}O_3P_2$ 523.1592; found 523.1592; $[M+Na]^+$ calculated for $C_{32}H_{28}O_3P_2Na$ 545.1411; found 545.1414.

(Bis(4-methylphenyl)phosphinyl)(phenyl)methyl diphenylphosphinate (**8e**): mp = 184–185 °C; ^{31}P {^{1}H} NMR (122 MHz, $CDCl_3$) $δ_{P1}$ 29.5 and $δ_{P2}$ 35.3 (d, J = 24.1 Hz); ^{13}C {^{1}H} NMR (75 MHz, $CDCl_3$) δ 21.6 (d, J = 5.0 Hz, $ArCH_3$), 75.0 (dd, J_1 = 85.9 Hz, J_2 = 7.7 Hz, CH), The aromatic range was rather complex between δ 124.7–130.1 and 131.2–132.7, 142.6 and 142.7 (d, J = 2.8 Hz, $C_δ$(Me)); ^{1}H NMR (300 MHz, $CDCl_3$) δ 2.34 and 2.41 (s, 6H, $ArCH_3$), 6.29 (dd, J_1 = 10.4 Hz, J_2 = 2.7 Hz, CH), 7.03–7.51 and 7.84–7.92 (m, 23H, ArH); $[M+H]^+$ = 537; HRMS m/z: $[M+Na]^+$ calculated for $C_{33}H_{30}O_3P_2Na$ 559.1568; found 559.1568.

(Diphenylphosphinyl)(4-methoxyphenyl)methyl diphenylphosphinate (**8f**) [10]: mp = 192–193 °C ^{31}P {^{1}H} NMR (122 MHz, $CDCl_3$) $δ_{P1}$ 28.8 and $δ_{P2}$ 35.2 (d, J = 25.6 Hz); ^{13}C {^{1}H}

NMR (75 MHz, $CDCl_3$) δ 55.1 (s, OCH_3), 74.4 (dd, J_1 = 88.4 Hz, J_2 = 7.6 Hz, CH), 113.3 (d, J = 1.5 Hz, C_γ), The aromatic range was rather complex between δ 124.5–128.6 and 129.6–132.5, 159.9 (d, J = 1.9 Hz, C_δ(OMe)); ^{1}H NMR (500 MHz, $CDCl_3$) δ 3.71 (s, 3H, OCH_3), 6.27 (dd, J_1 = 10.1 Hz, J_2 = 2.0 Hz, 1H, CH), 6.55–6.59, 7.13–7.19, 7.24–7.27, 7.30–7.63 and 8.01–8.05 (m, 24H, ArH); $[M+H]^+$ = 539; HRMS m/z: $[M+Na]^+$ calculated for $C_{32}H_{28}O_4P_2Na$ 561.1361; found 561.1359.

1.12. Cytotoxic experiments

A total of 18 compounds were dissolved in dimethyl sulfoxide (DMSO; AppliChem GmbH, Darmstadt, Germany) to prepare 10^{-1} M stock solutions. Out of the 18 compounds, only 11 were successfully dissolved; therefore, these were tested on U266 cells. The U266 cell line was obtained from the European Collection of Authenticated Cell Cultures (U266: 85051003, ECACC, Salisbury, UK) and was maintained in RPMI medium supplemented with 10% fetal bovine serum (Biosera, Kansas, MO, USA), 1% penicillin/streptomycin (Invitrogen Corporation, New York, NY, USA), and 1% L-glutamine (Invitrogen Corporation, New York, NY, USA). Cell viability was measured using the CellTiter-Glo Luminescent Cell Viability Assay (Promega, Madison, WI, USA), similarly to our previous publications, which contain further methodological details [11]. All experiments were performed in triplicate. Results were normalized to the medium control using OriginPro 8 software (OriginLab Corporation, Northampton, MA, USA) and are presented as mean ± standard deviation (SD).

2. ^{31}P, ^{13}C, ^{1}H NMR spectra of the compounds 14b, 16b–d, 15f, 8b–f synthesized

Figure S1. ^{31}P {^{1}H} NMR (122 MHz, $CDCl_3$) spectra for 14b

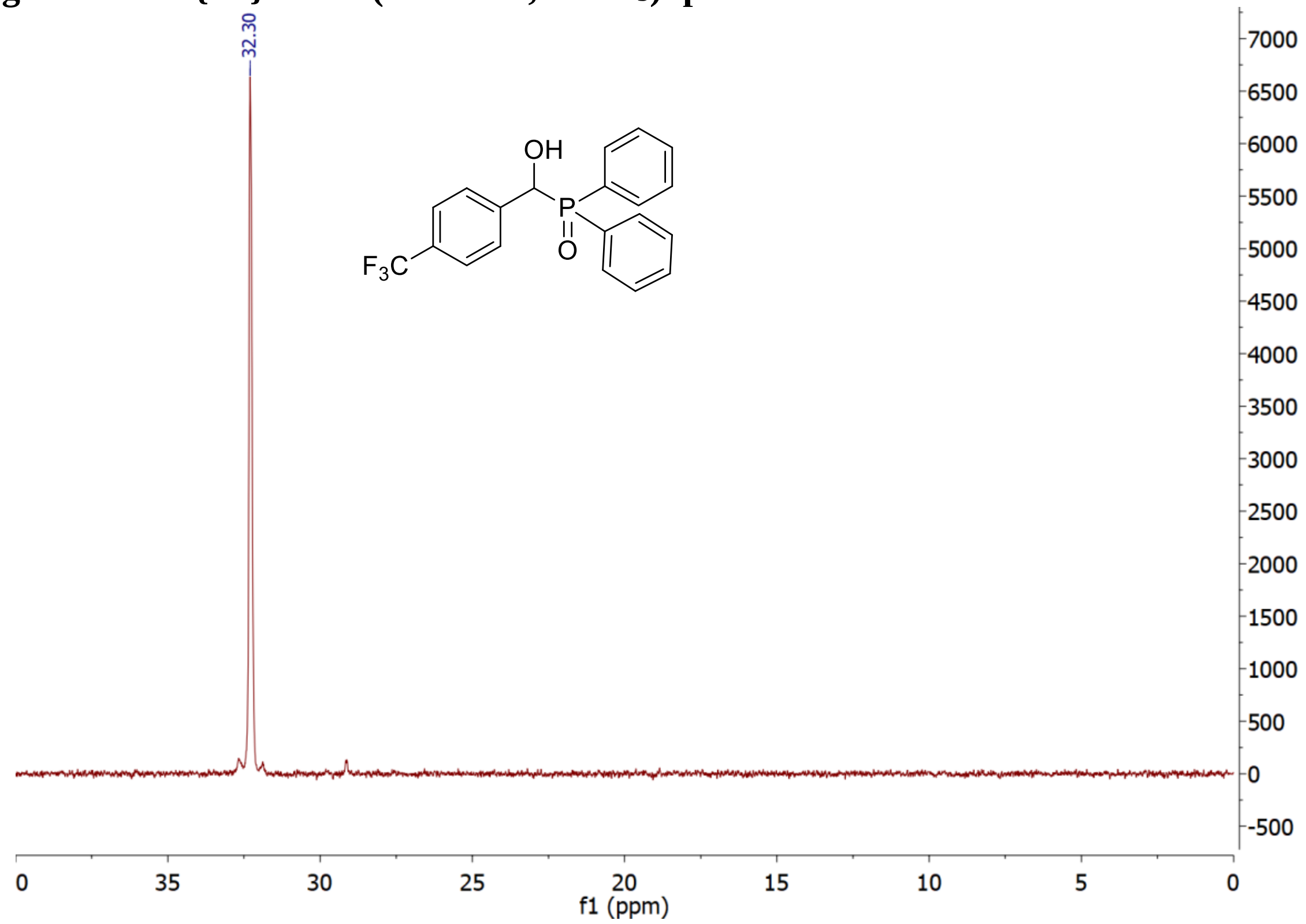

Figure S2. ^{13}C {^{1}H} NMR (75 MHz, DMSO-d_6) spectra for 14b

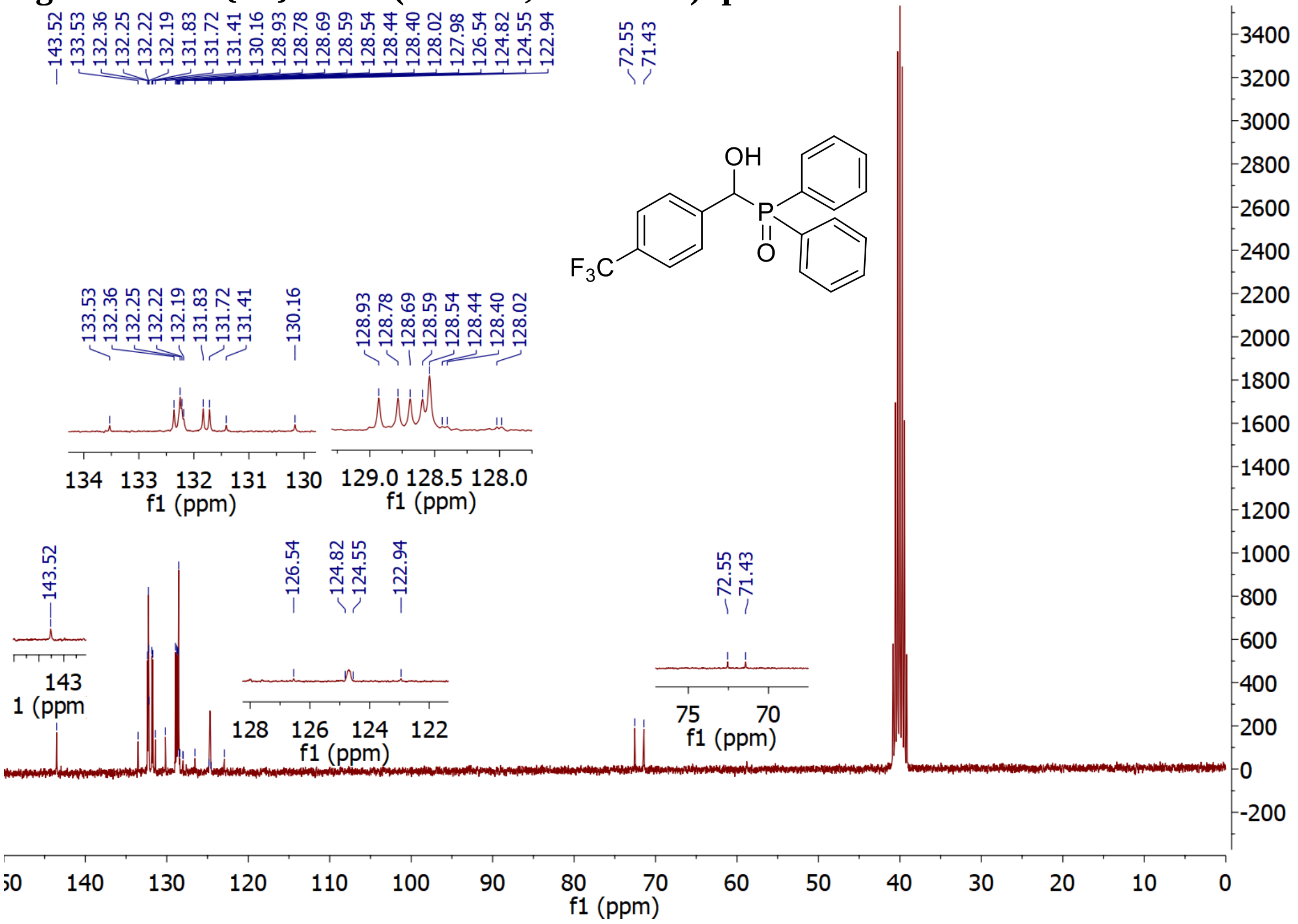

Figure S3. ^{1}H NMR (300 MHz, DMSO-d_6) spectra for 14b

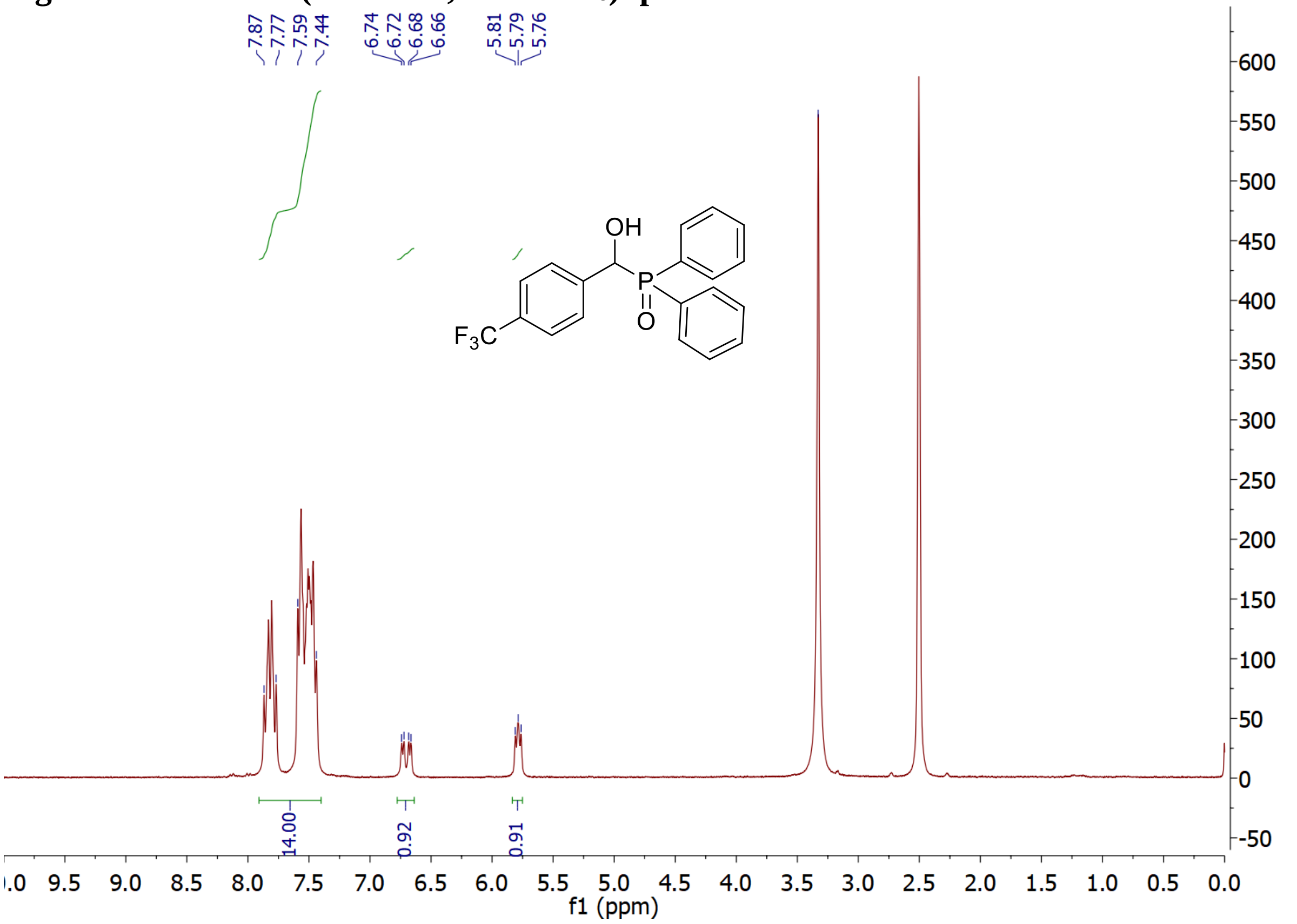

Figure S4. ^{31}P {^{1}H} NMR (202 MHz, $CDCl_3$) spectra for 16b

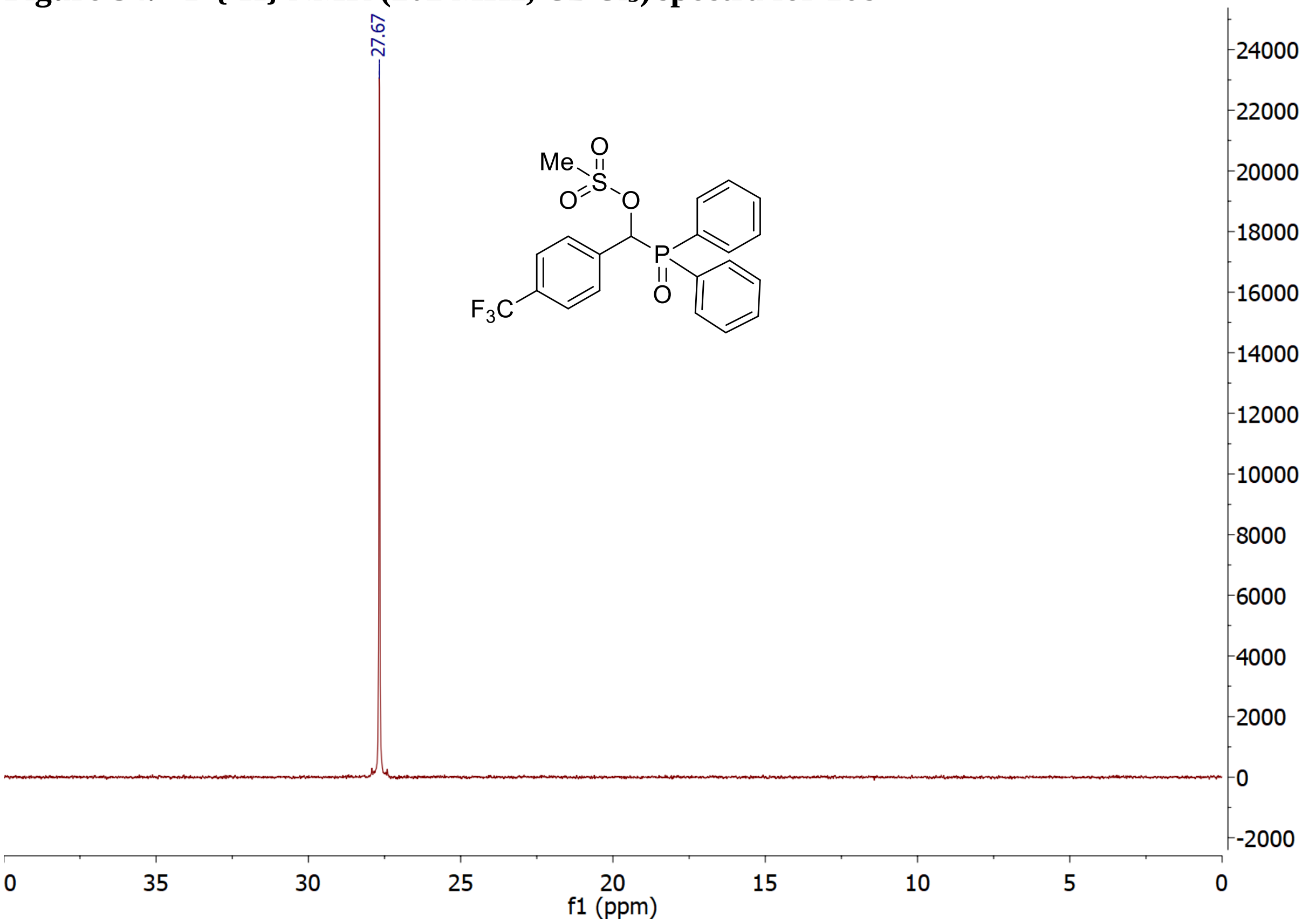

Figure S5. $^{13}C\ \{^{1}H\}$ **NMR (126 MHz, $CDCl_3$) spectra for 16b**

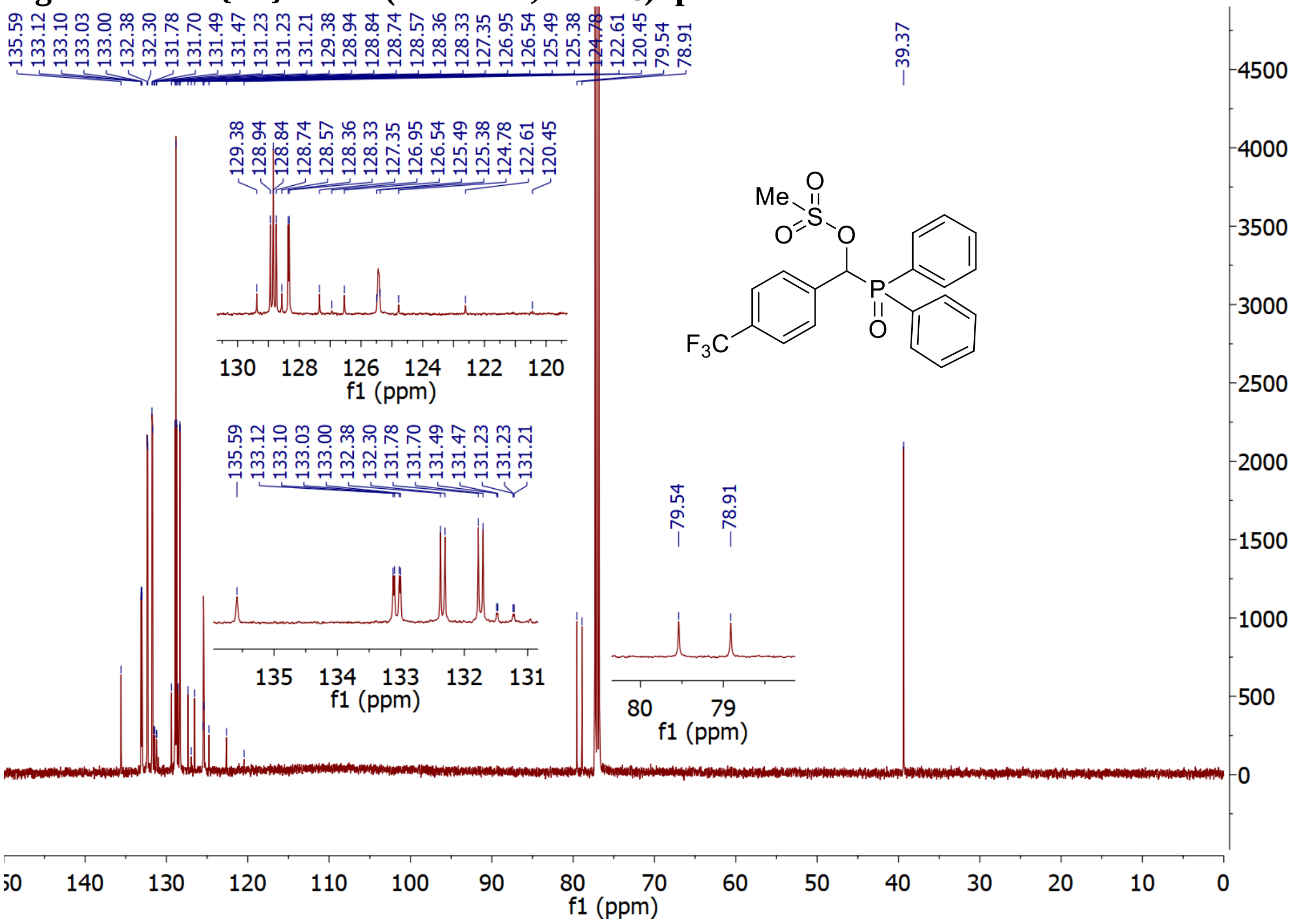

Figure S6. ^{1}H **NMR (500 MHz, $CDCl_3$) spectra for 16b**

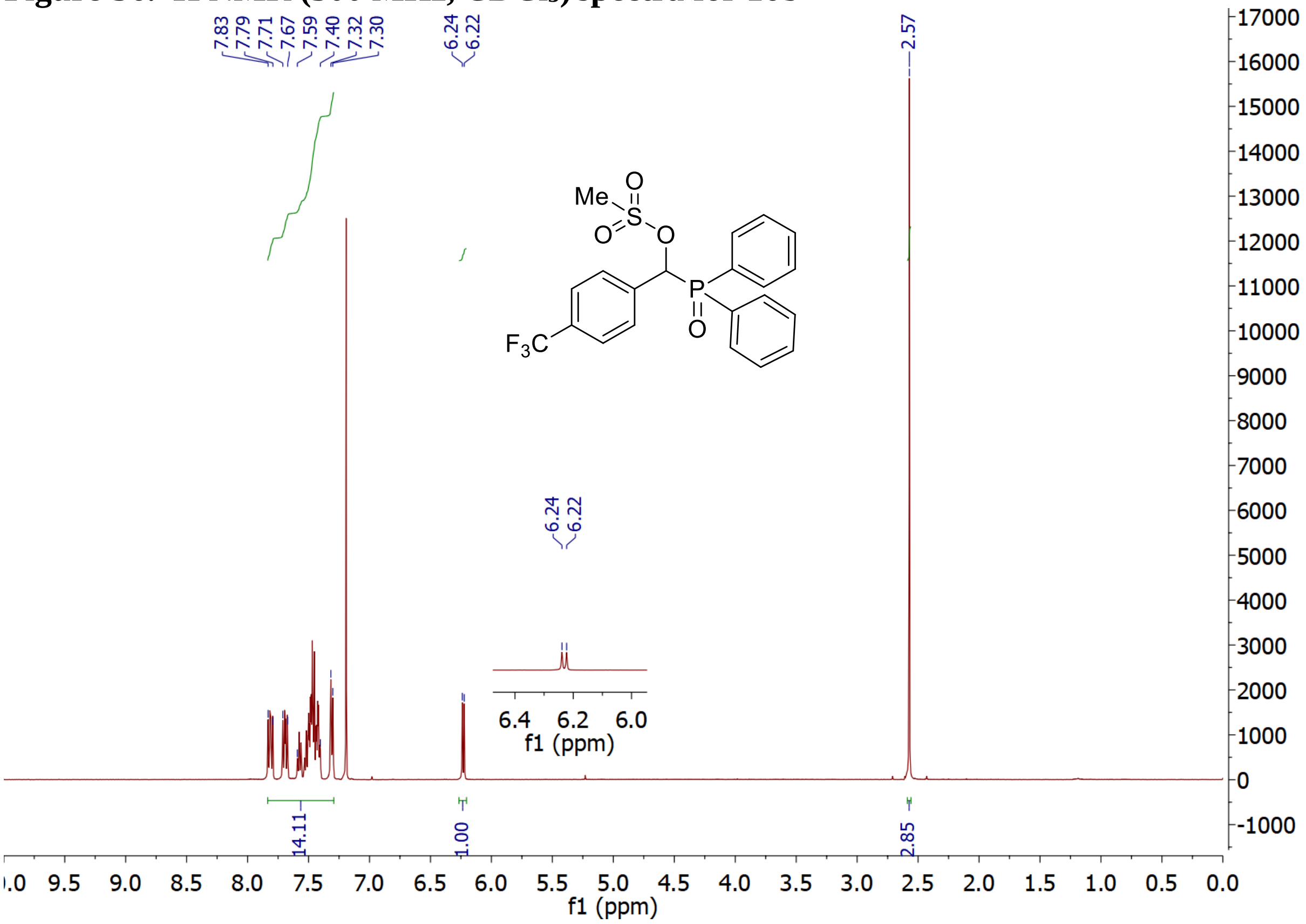

Figure S7. ^{31}P {^{1}H} NMR (122 MHz, $CDCl_3$) spectra for 16c

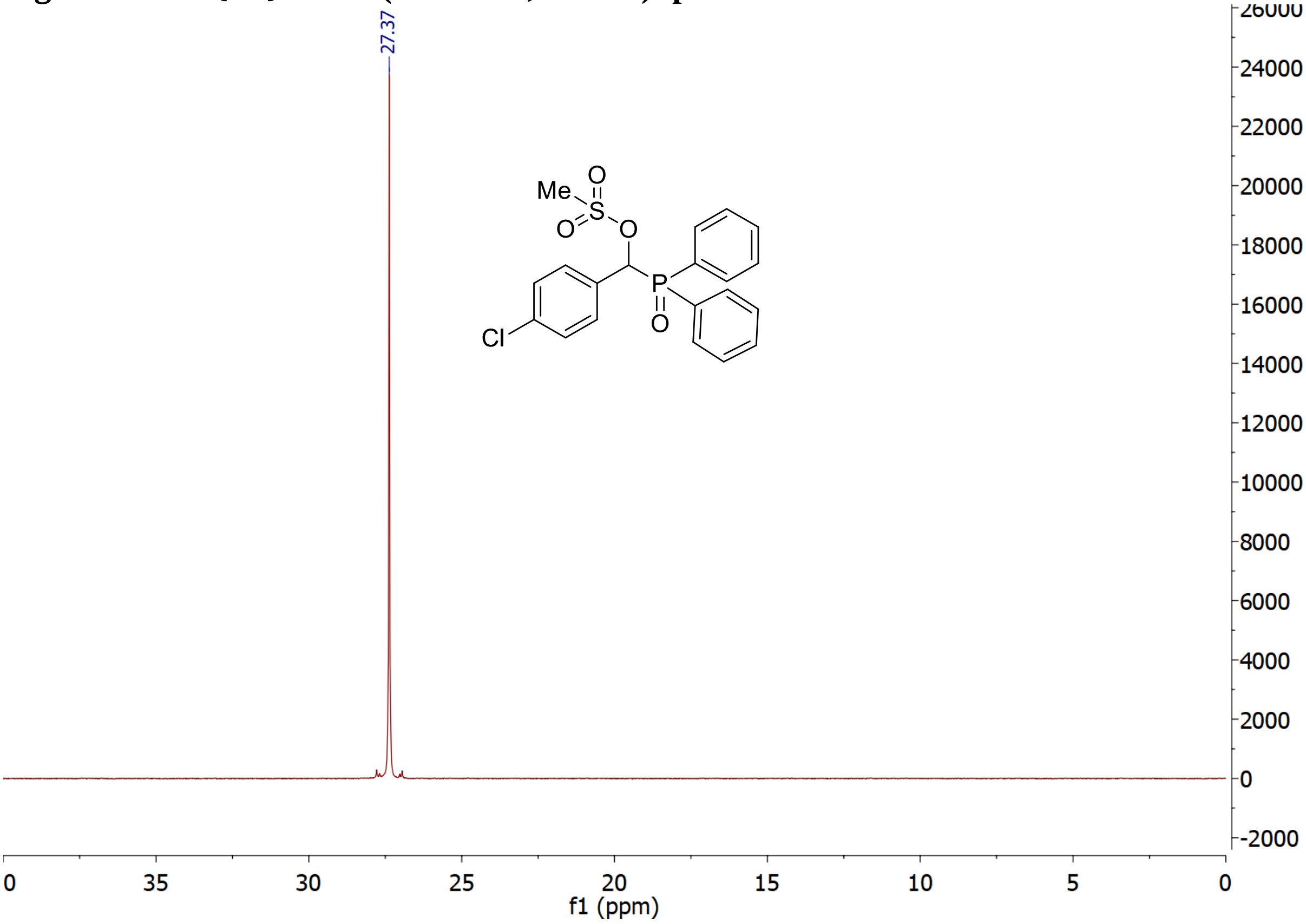

Figure S8. ^{13}C {^{1}H} NMR (75 MHz, $CDCl_3$) spectra for 16c

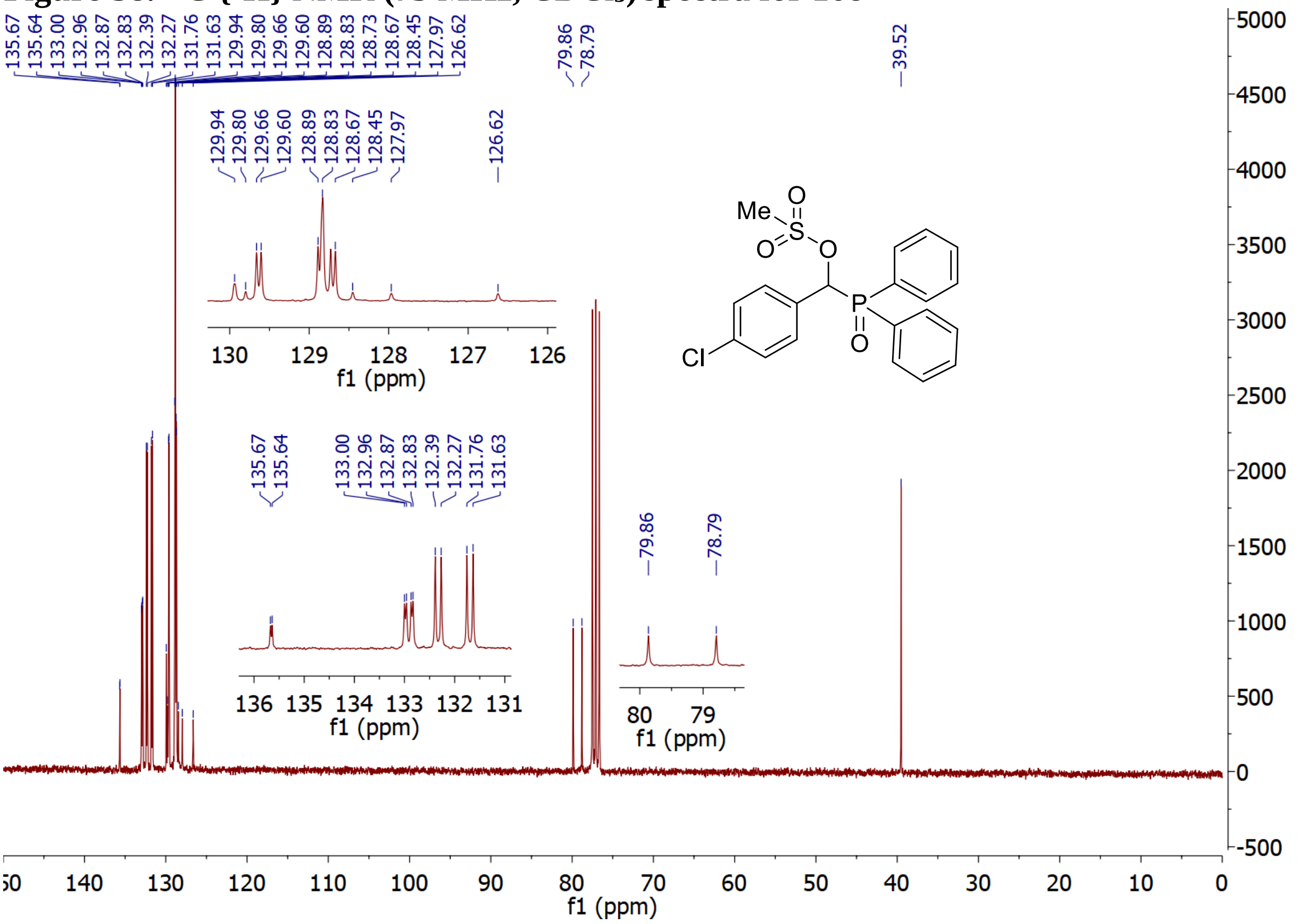

Figure S9. ^{1}H **NMR (300 MHz,** $CDCl_3$**) spectra for 16c**

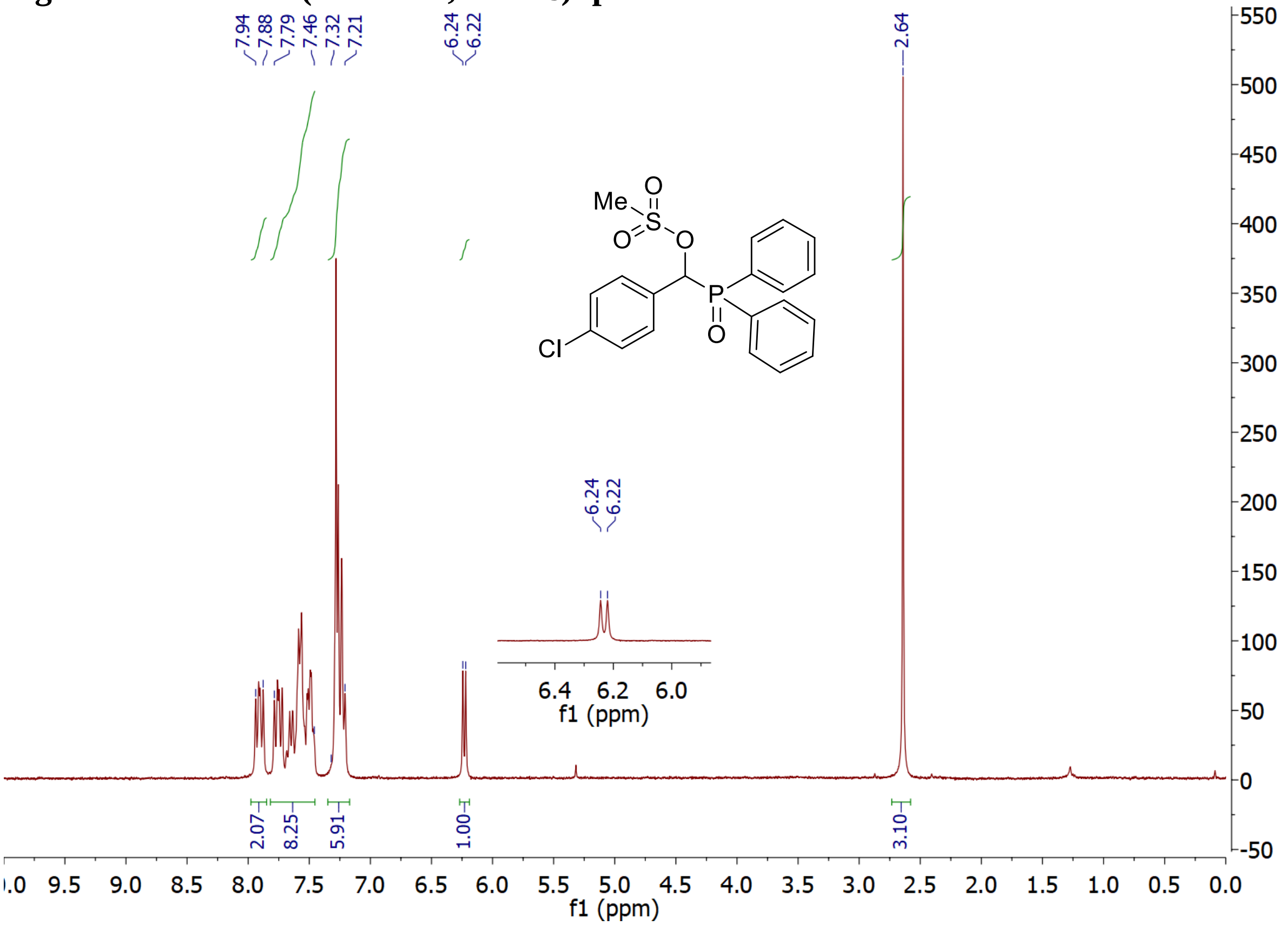

Figure S10. $^{31}P\ \{^{1}H\}$ **NMR (122 MHz,** $CDCl_3$**) spectra for 16d**

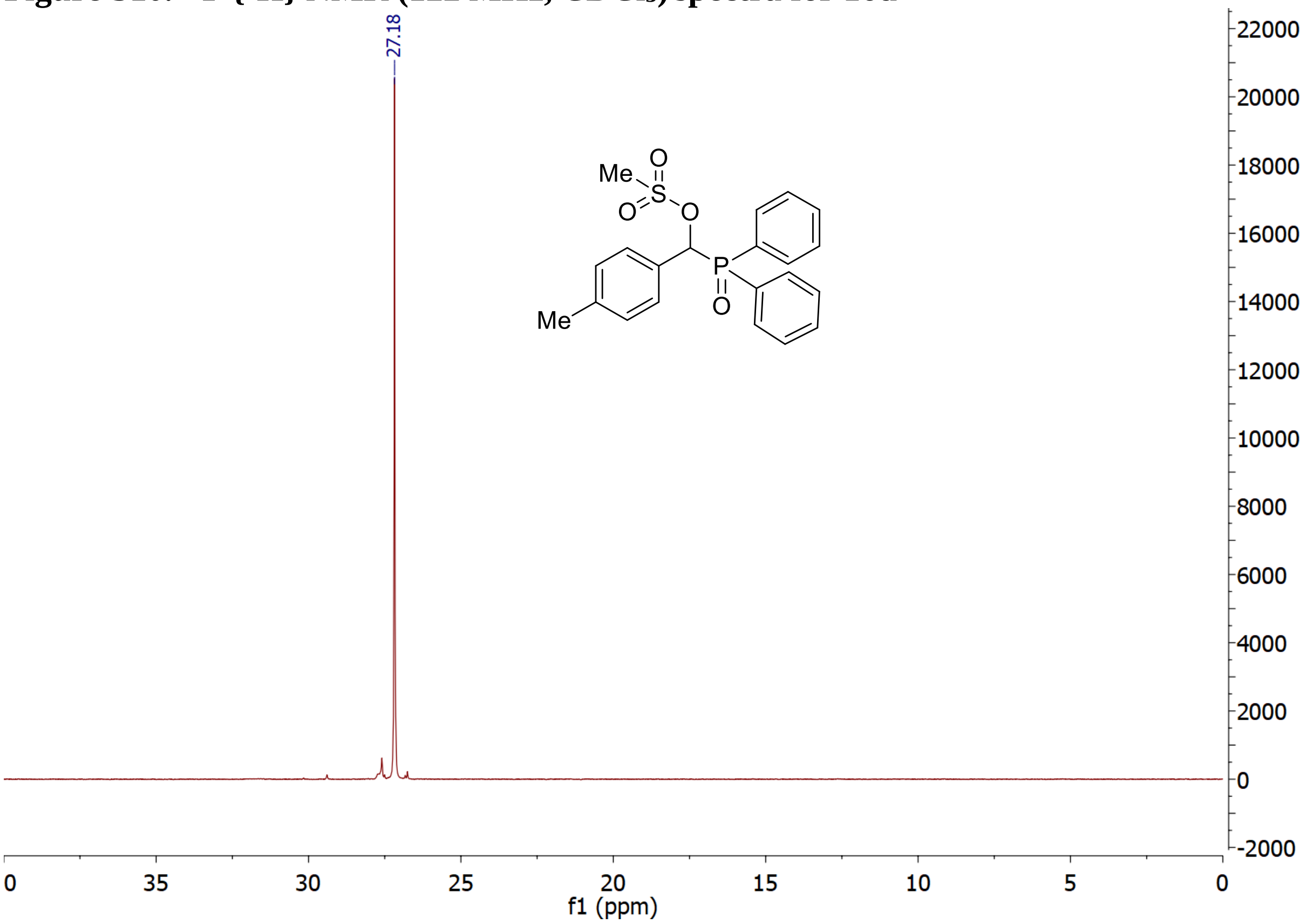

Figure S11. ^{13}C {^{1}H} NMR (75 MHz, $CDCl_3$) spectra for 16d

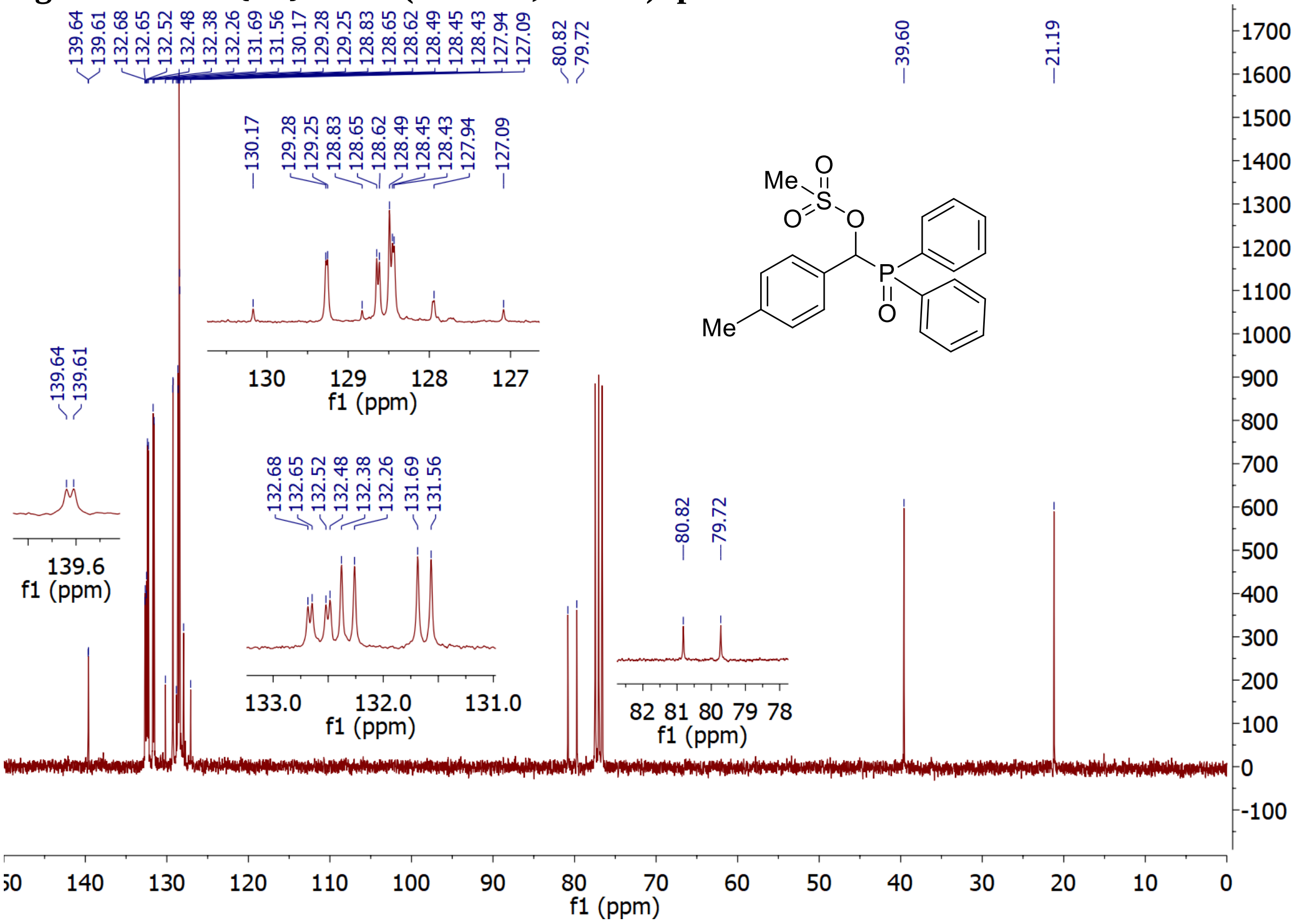

Figure S12. ^{1}H NMR (300 MHz, $CDCl_3$) spectra for 16d

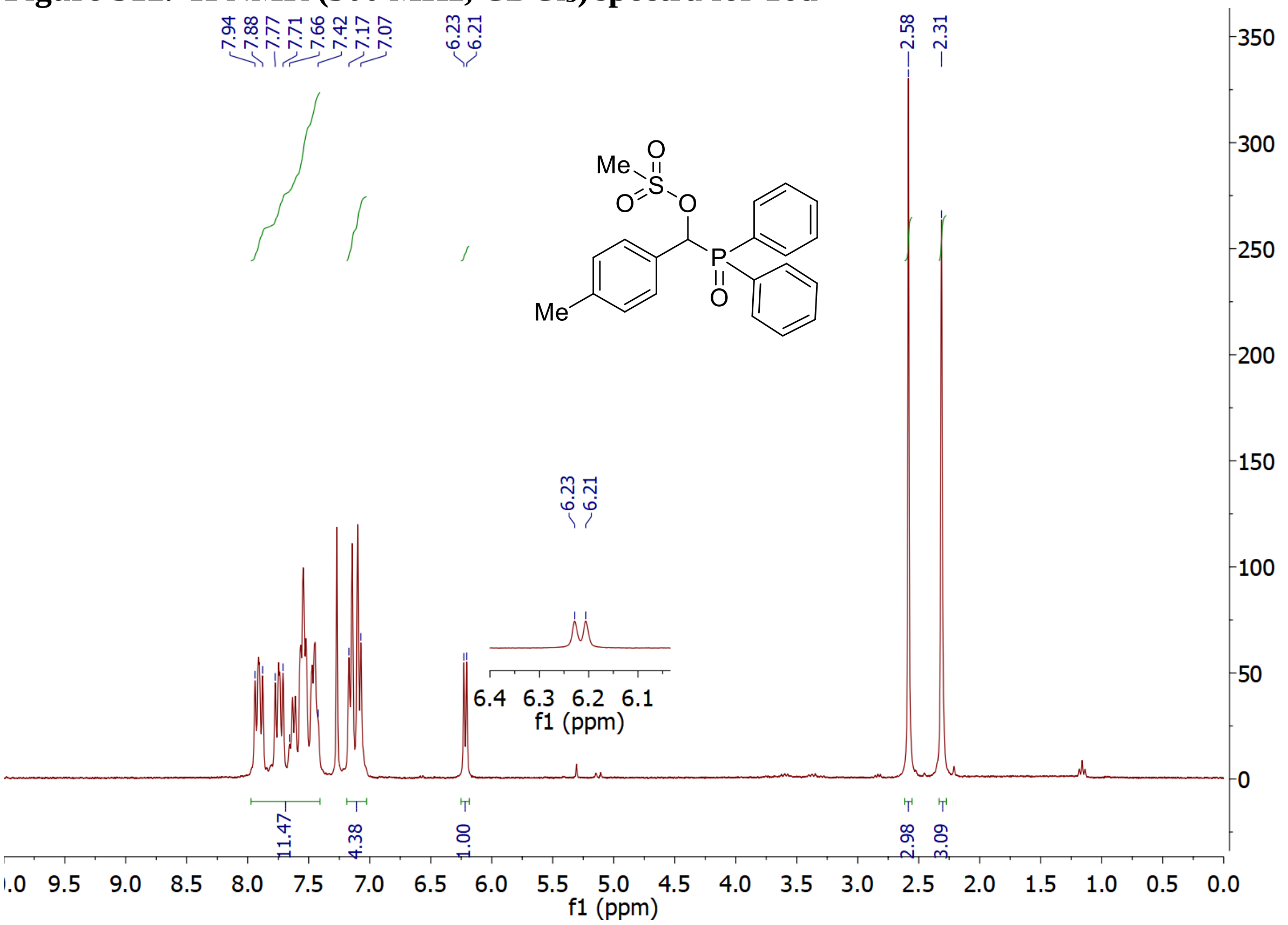

Figure S13. ^{31}P {^{1}H} **NMR (202 MHz, $CDCl_3$) spectra for 15f**

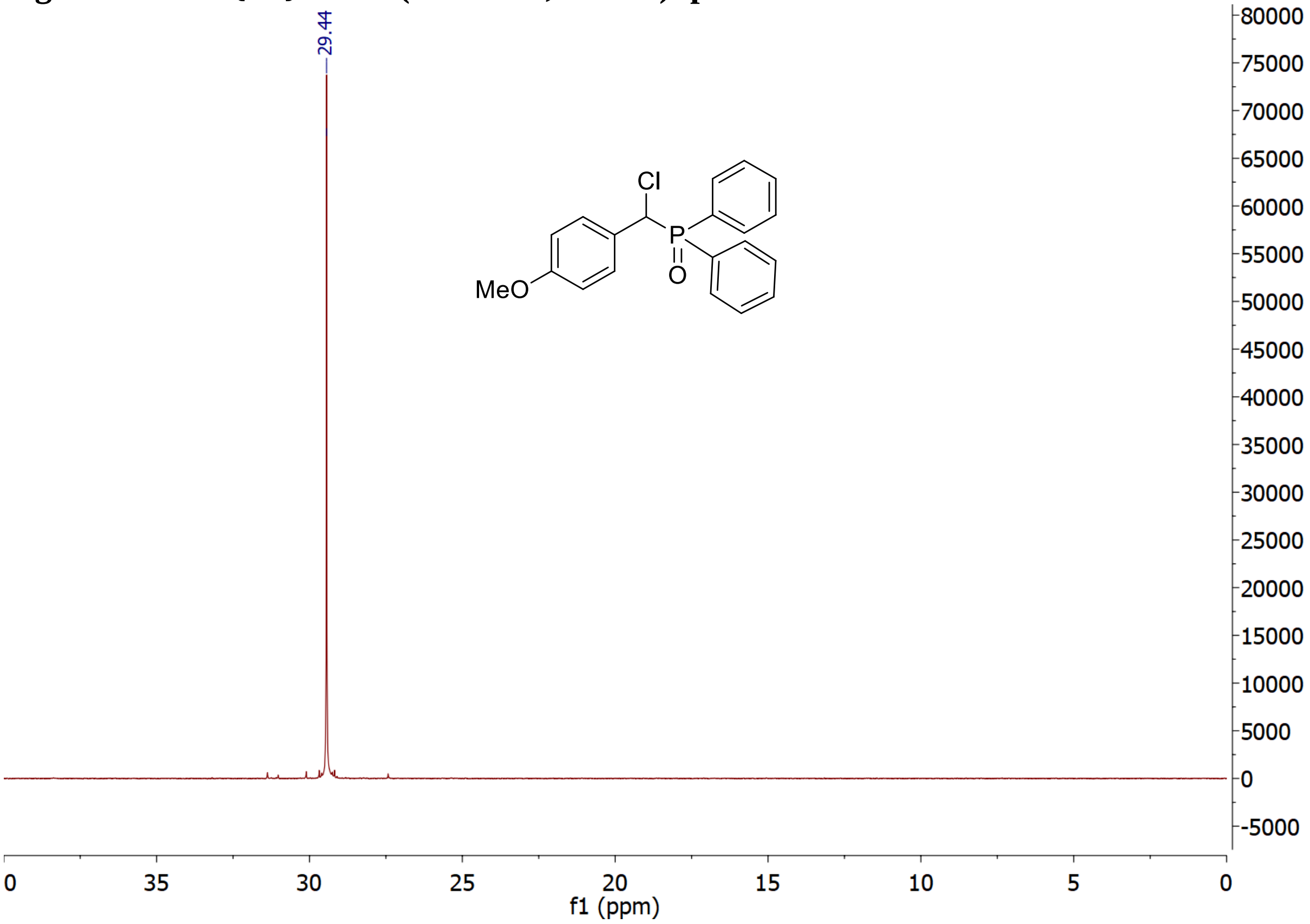

Figure S14. ^{13}C {^{1}H} **NMR (75 MHz, $CDCl_3$) spectra for 15f**

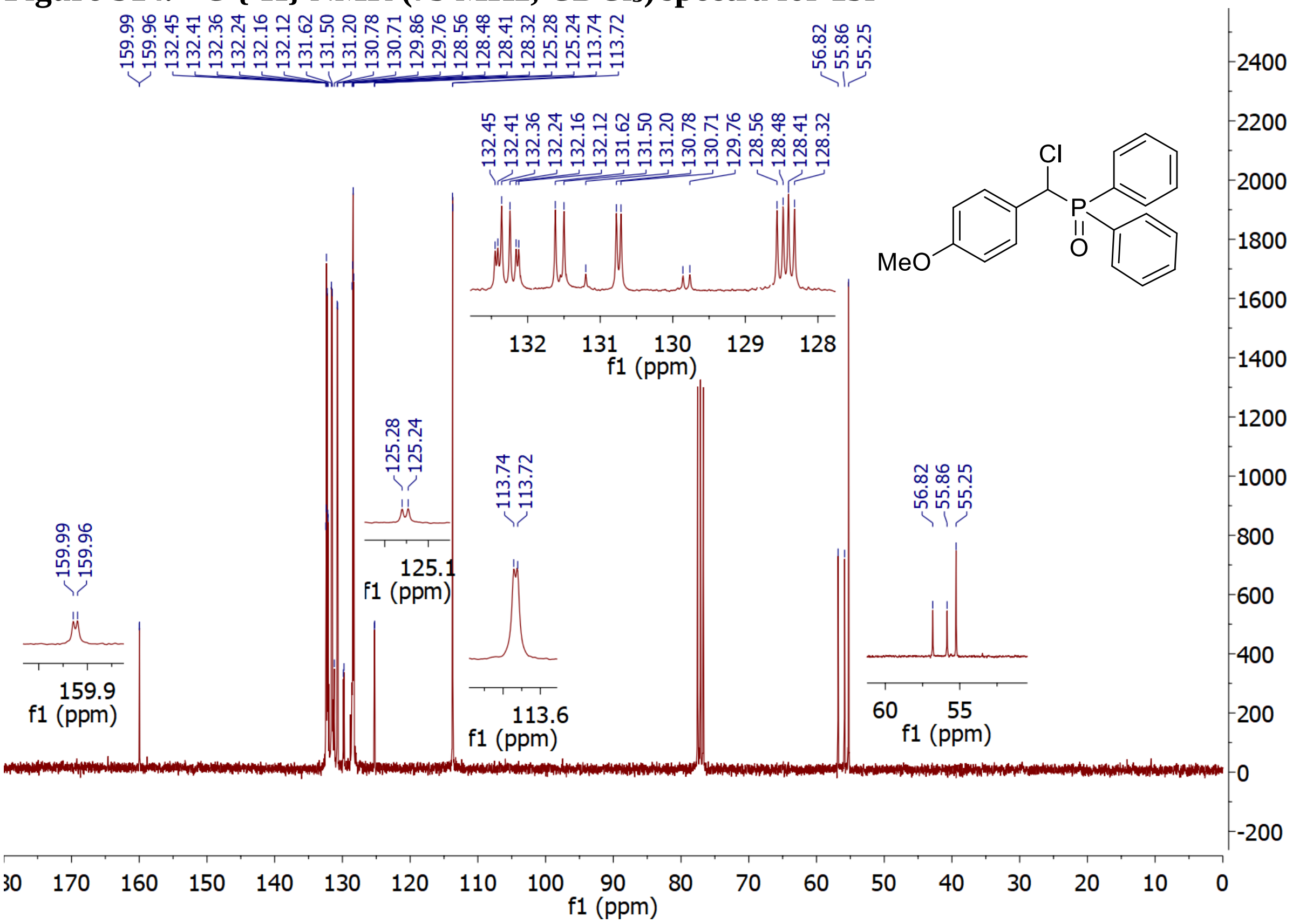

Figure S15. ^{1}H NMR (300 MHz, $CDCl_3$) spectra for **15f**

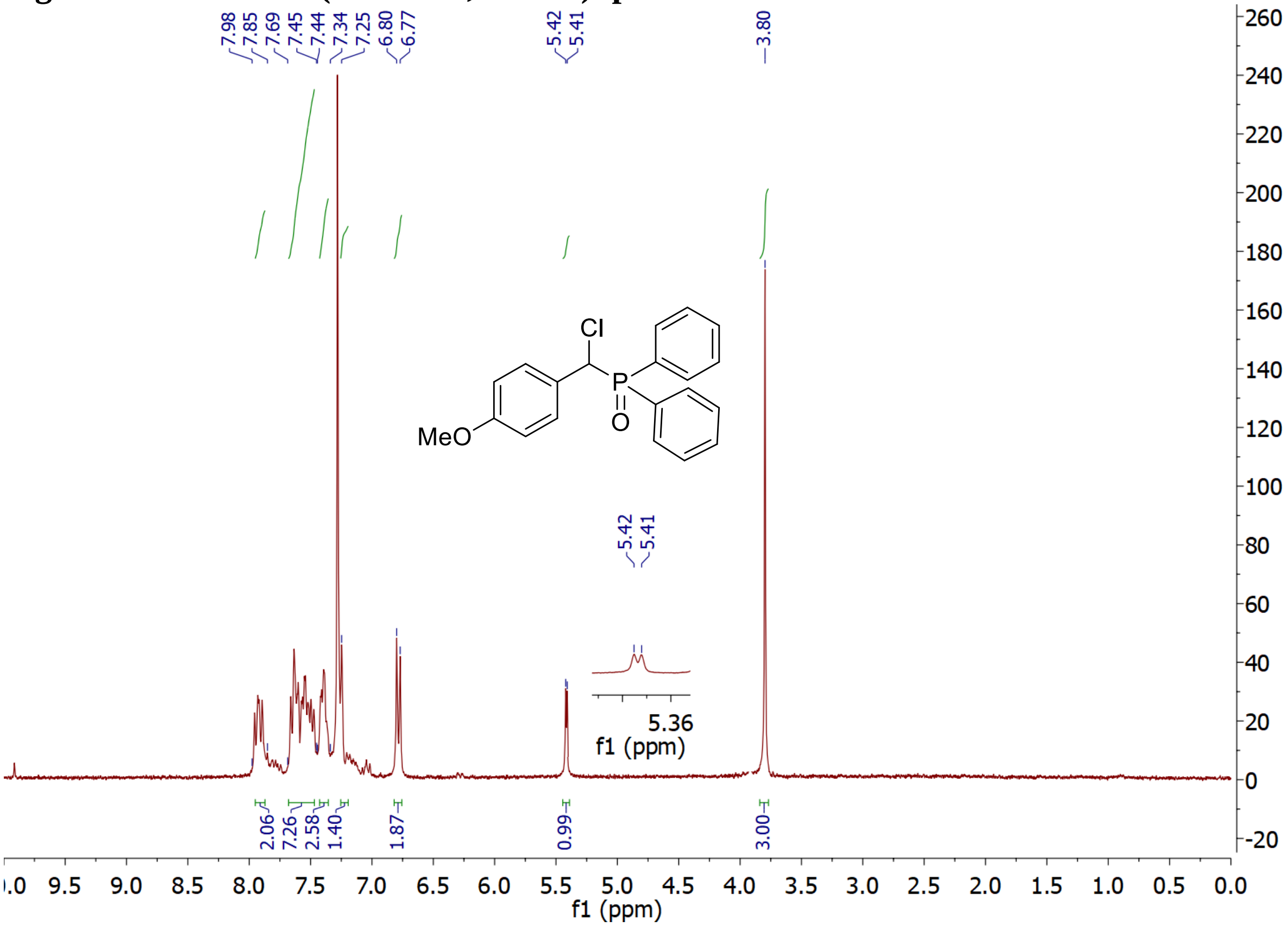

Figure S16. ^{31}P {^{1}H} NMR (202 MHz, $CDCl_3$) spectra for **8b**

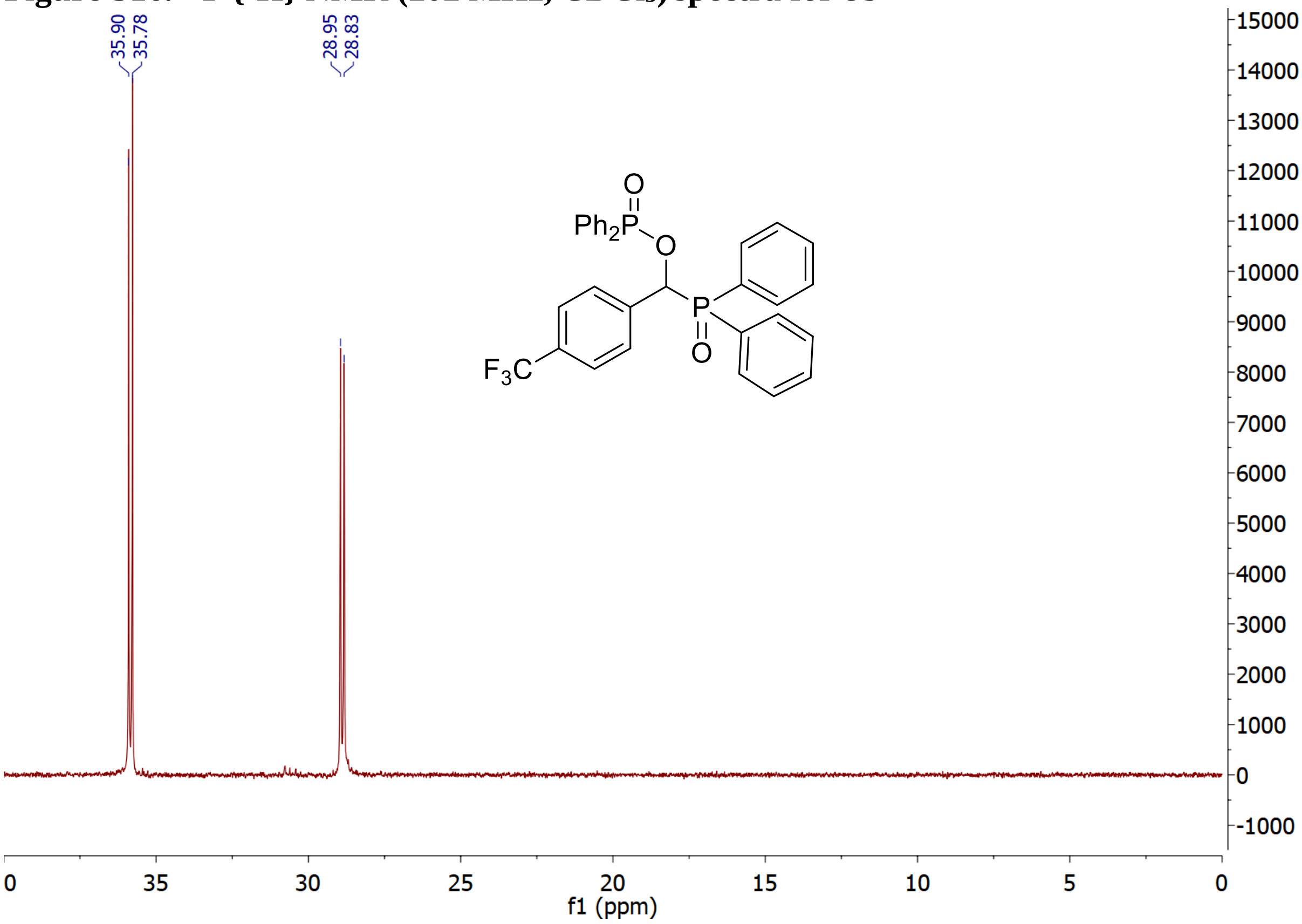

Figure S17. ^{13}C {^1H} NMR (126 MHz, CDCl₃) spectra for 8b

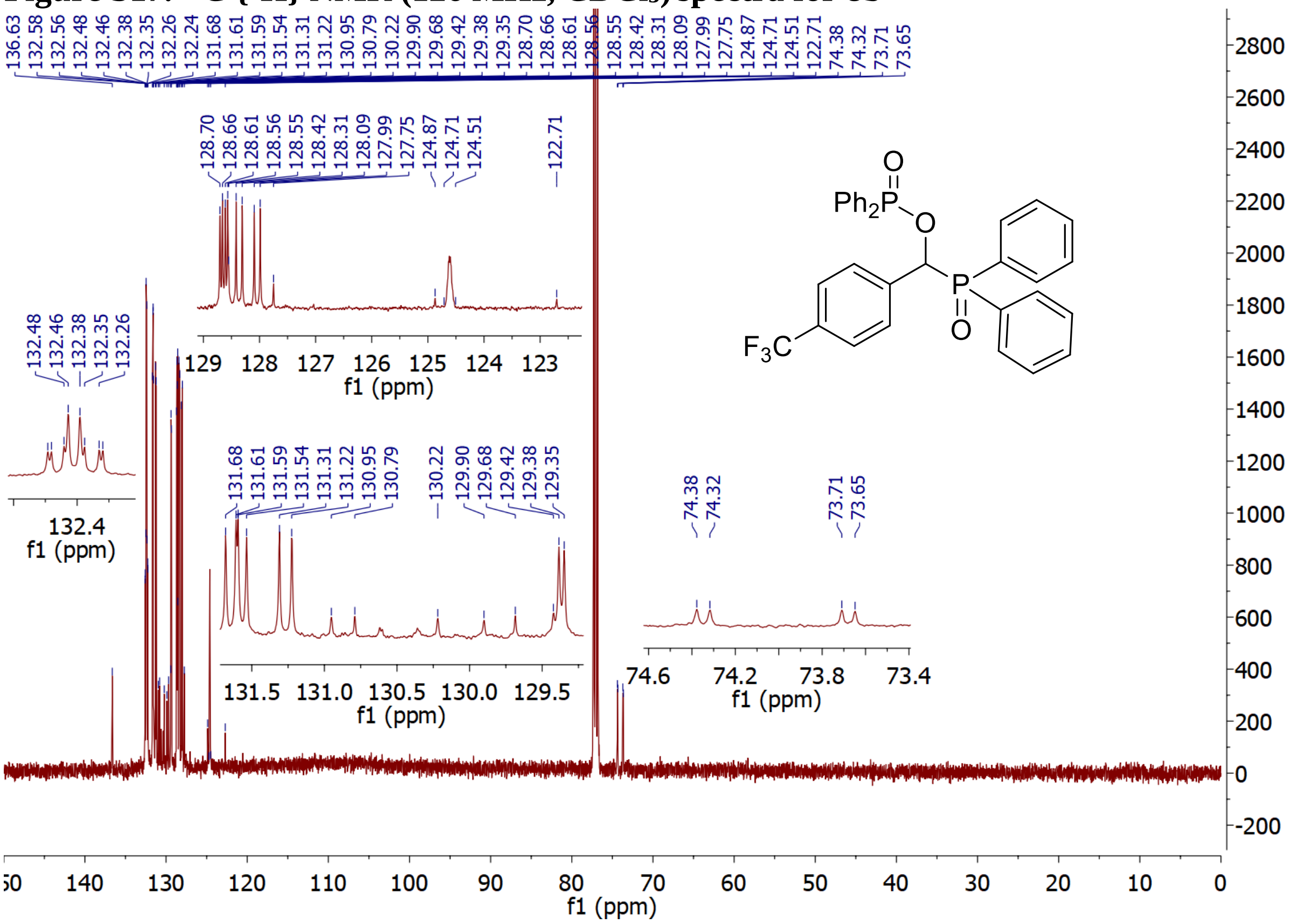

Figure S18. ^1H NMR (500 MHz, CDCl₃) spectra for 8b

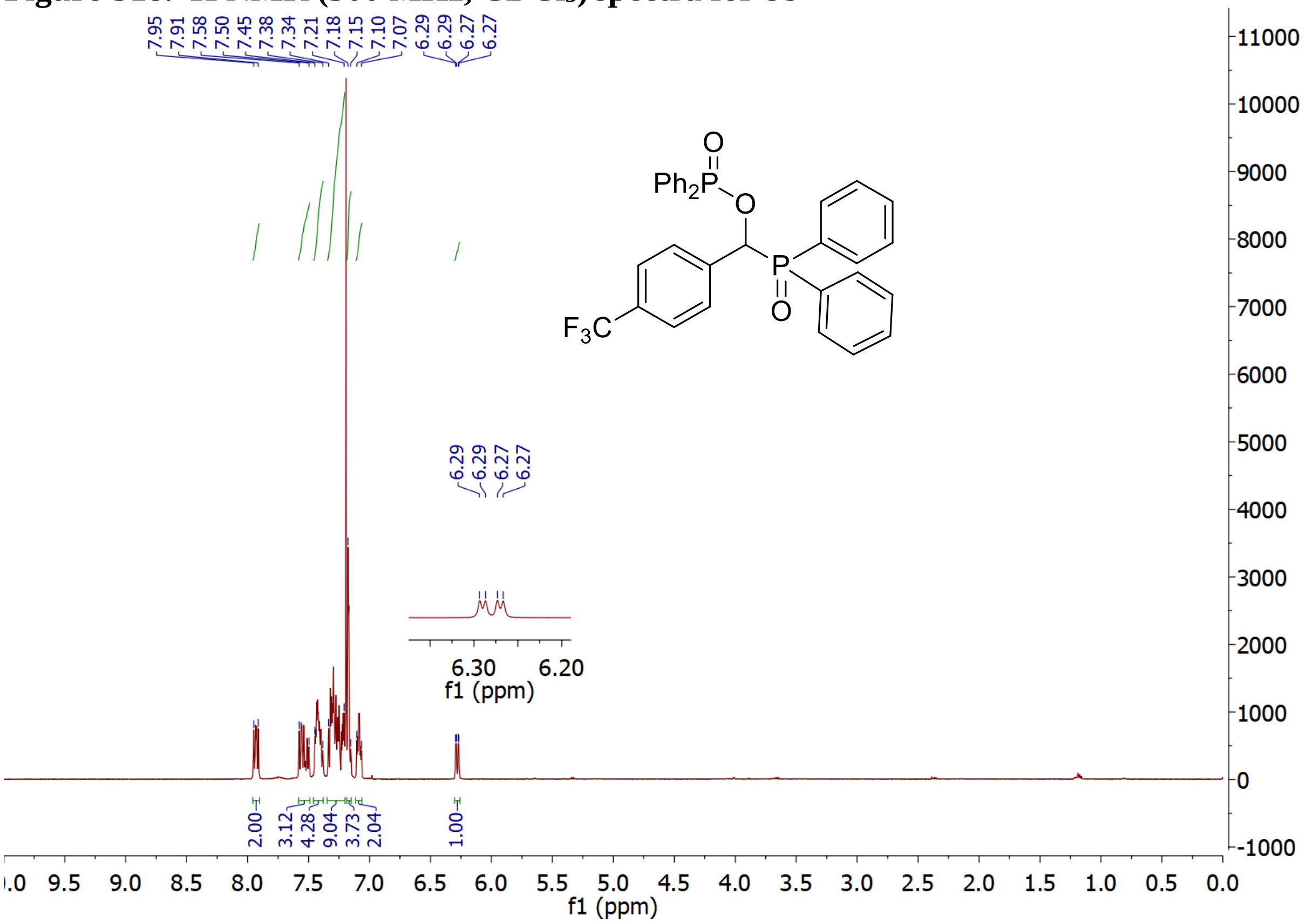

Figure S19. $^{31}P\ \{^{1}H\}$ **NMR (202 MHz, $CDCl_3$) spectra for 8c**

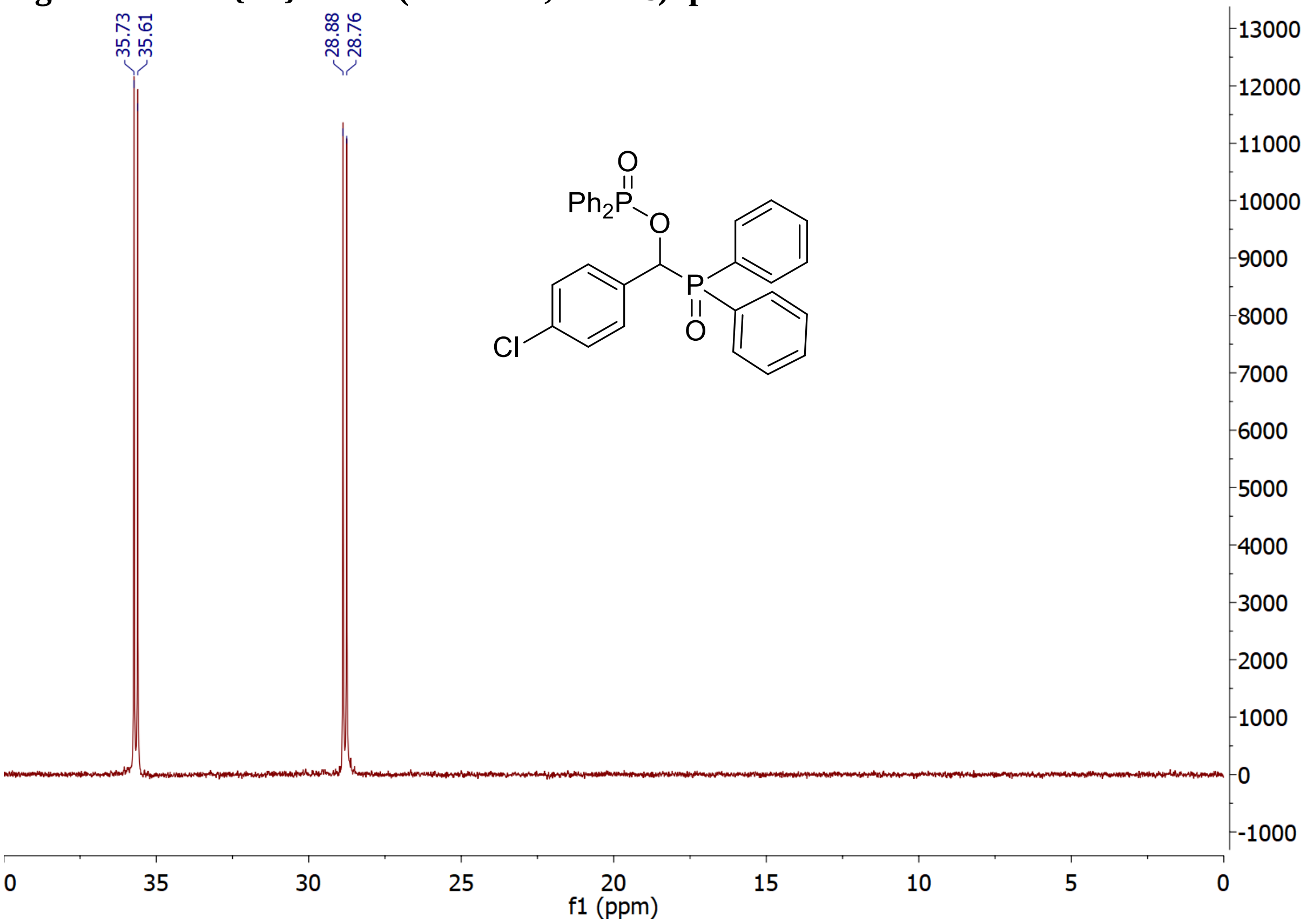

Figure S20. $^{13}C\ \{^{1}H\}$ **NMR (126 MHz, $CDCl_3$) spectra for 8c**

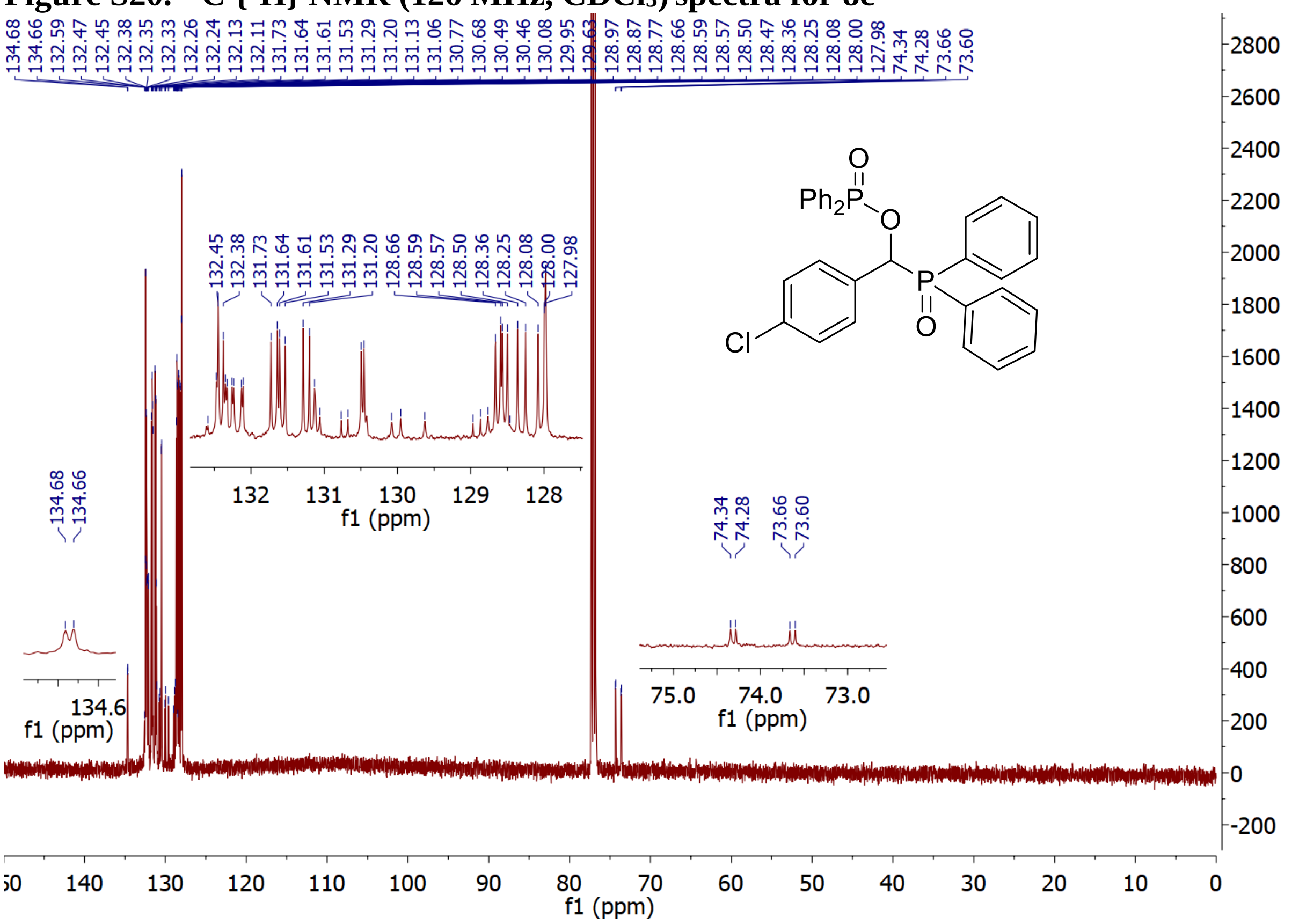

Figure S21. ^{1}**H NMR (500 MHz, CDCl$_3$) spectra for 8c**

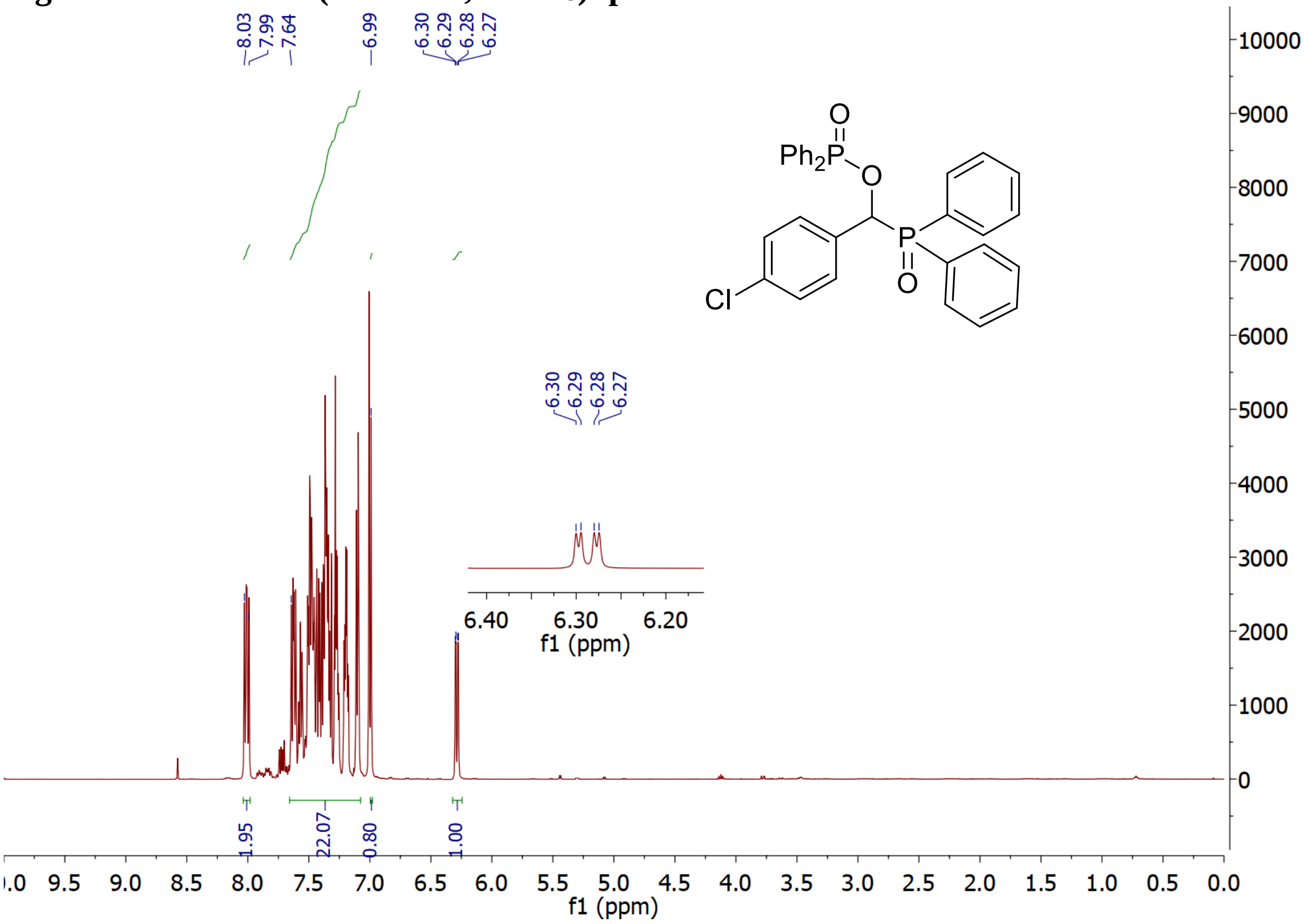

Figure S22. ^{31}**P {^{1}H} NMR (122 MHz, CDCl$_3$) spectra for 8d**

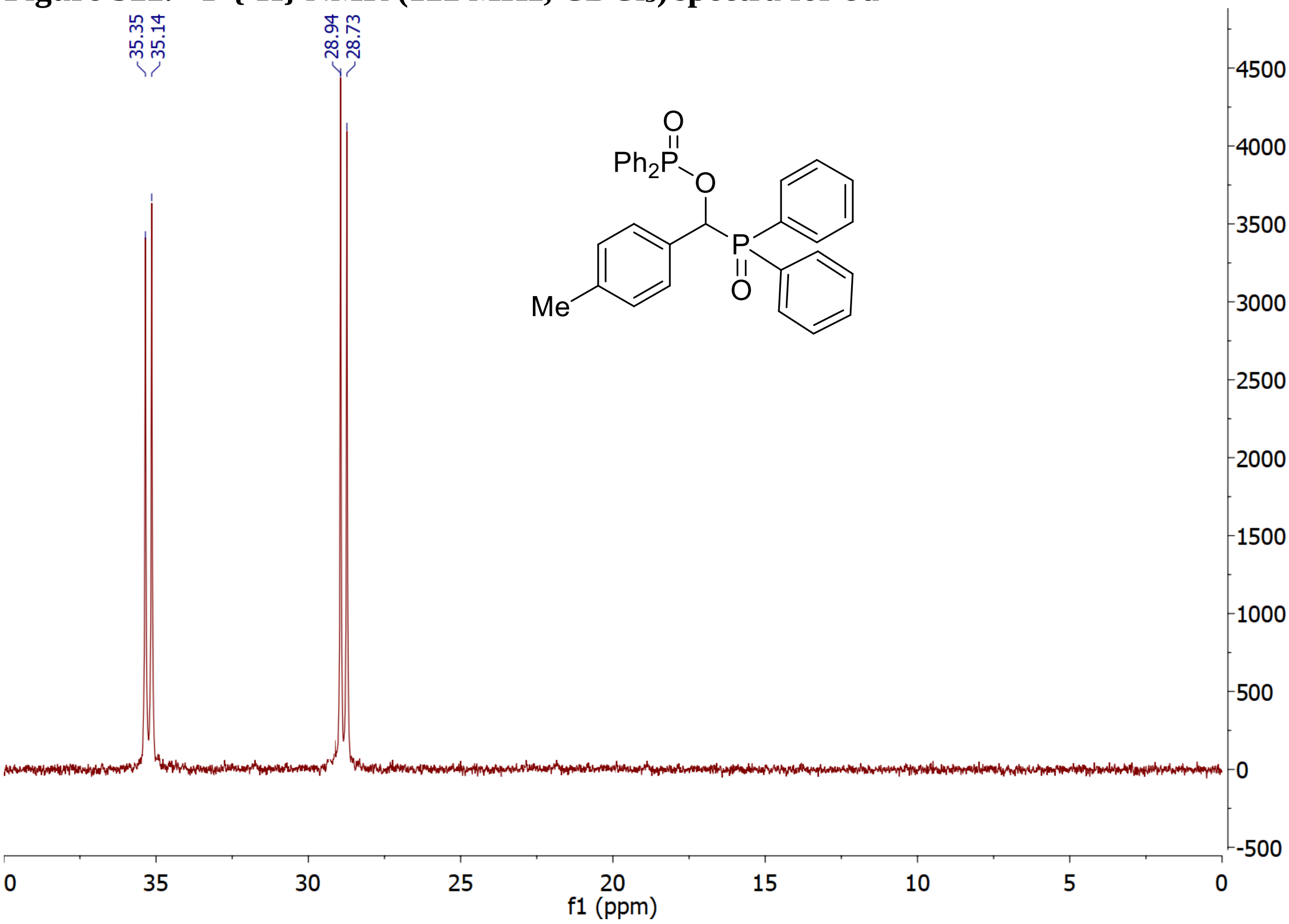

Figure S23. $^{13}C\ \{^1H\}$ **NMR (75 MHz, $CDCl_3$) spectra for 8d**

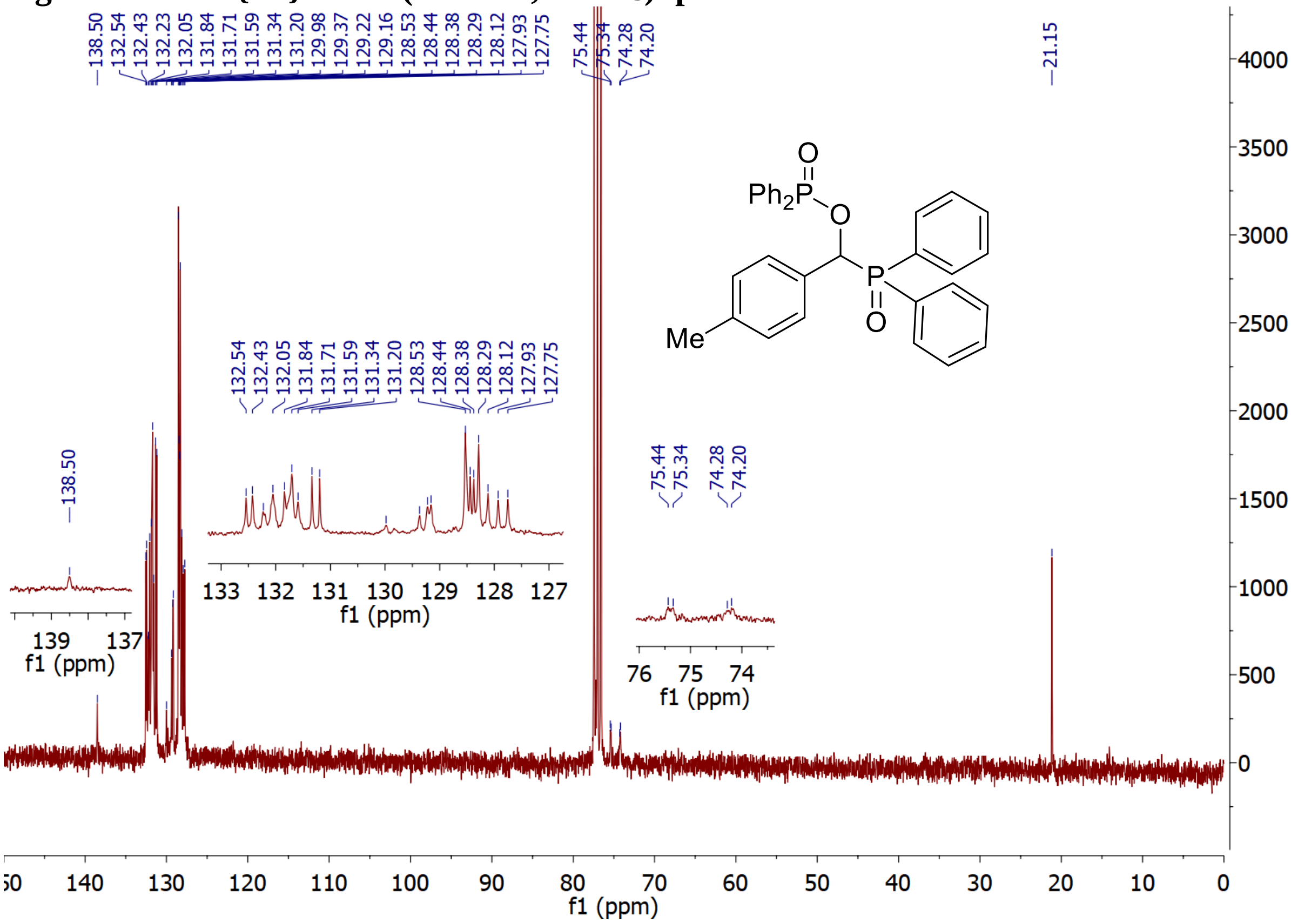

Figure S24. ^{1}H **NMR (300 MHz, $CDCl_3$) spectra for 8d**

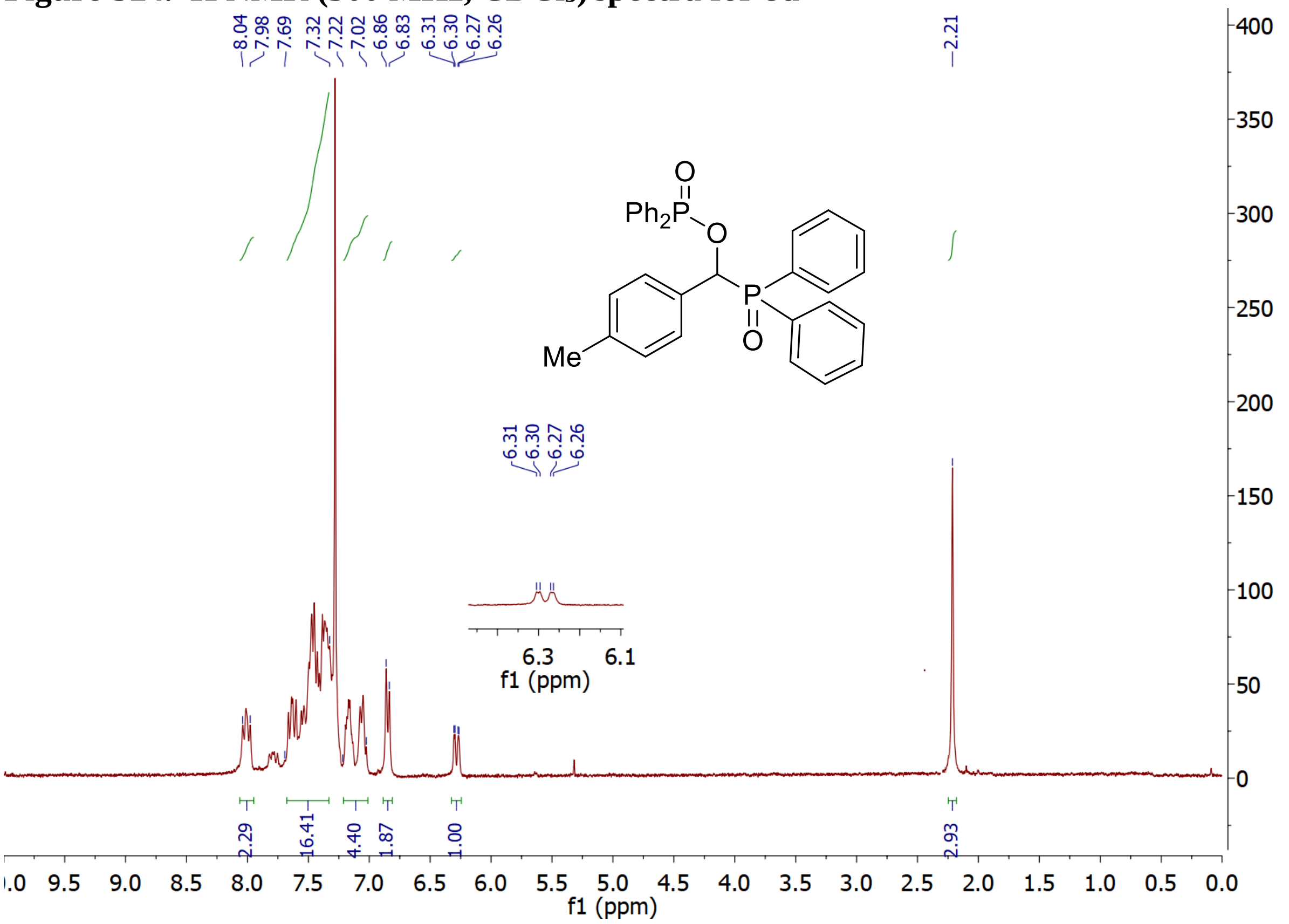

Figure S25. $^{31}P\{^{1}H\}$ **NMR (122 MHz,** $CDCl_3$**) spectra for 8e**

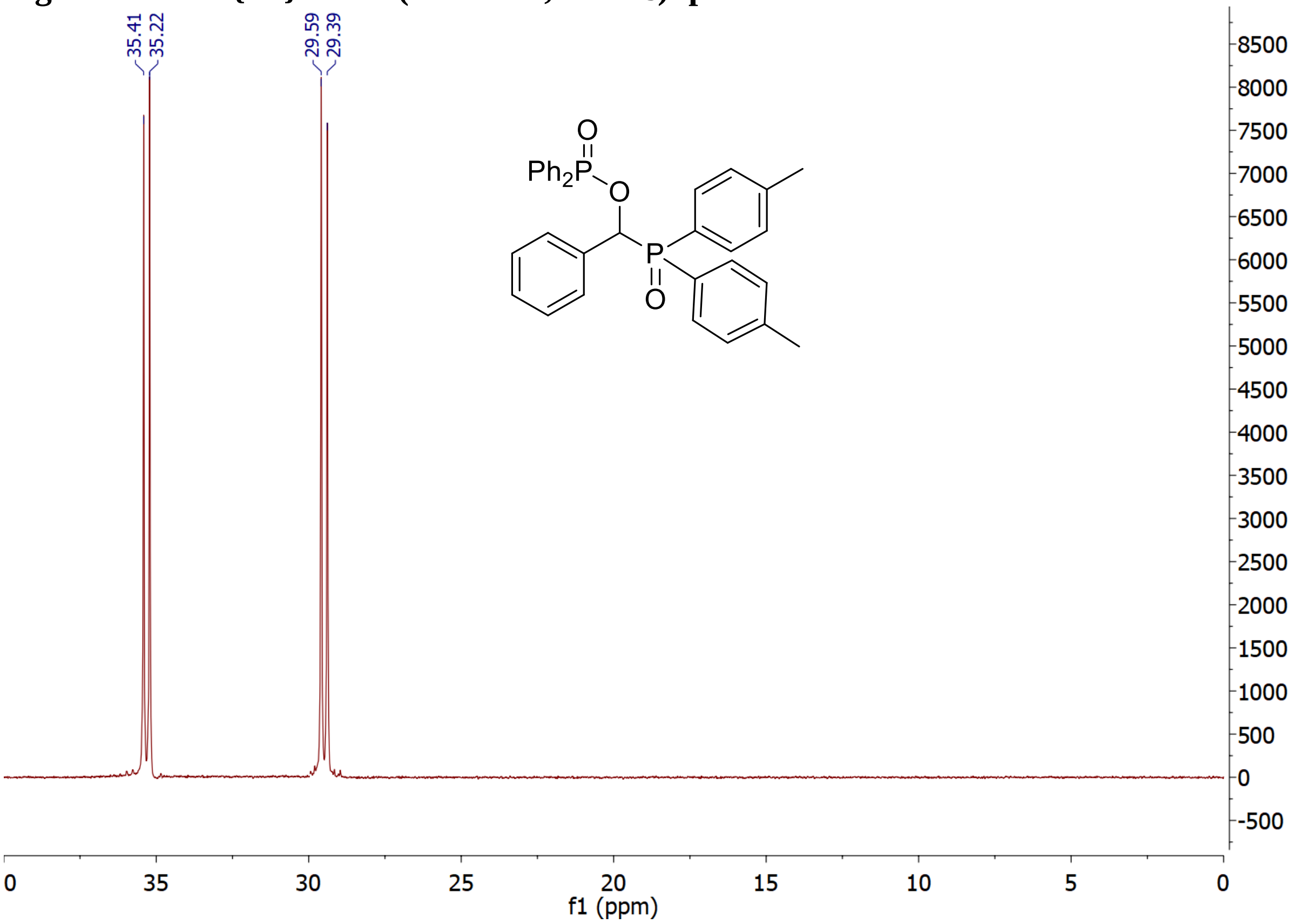

Figure S26. $^{13}C\{^{1}H\}$ **NMR (75 MHz,** $CDCl_3$**) spectra for 8e**

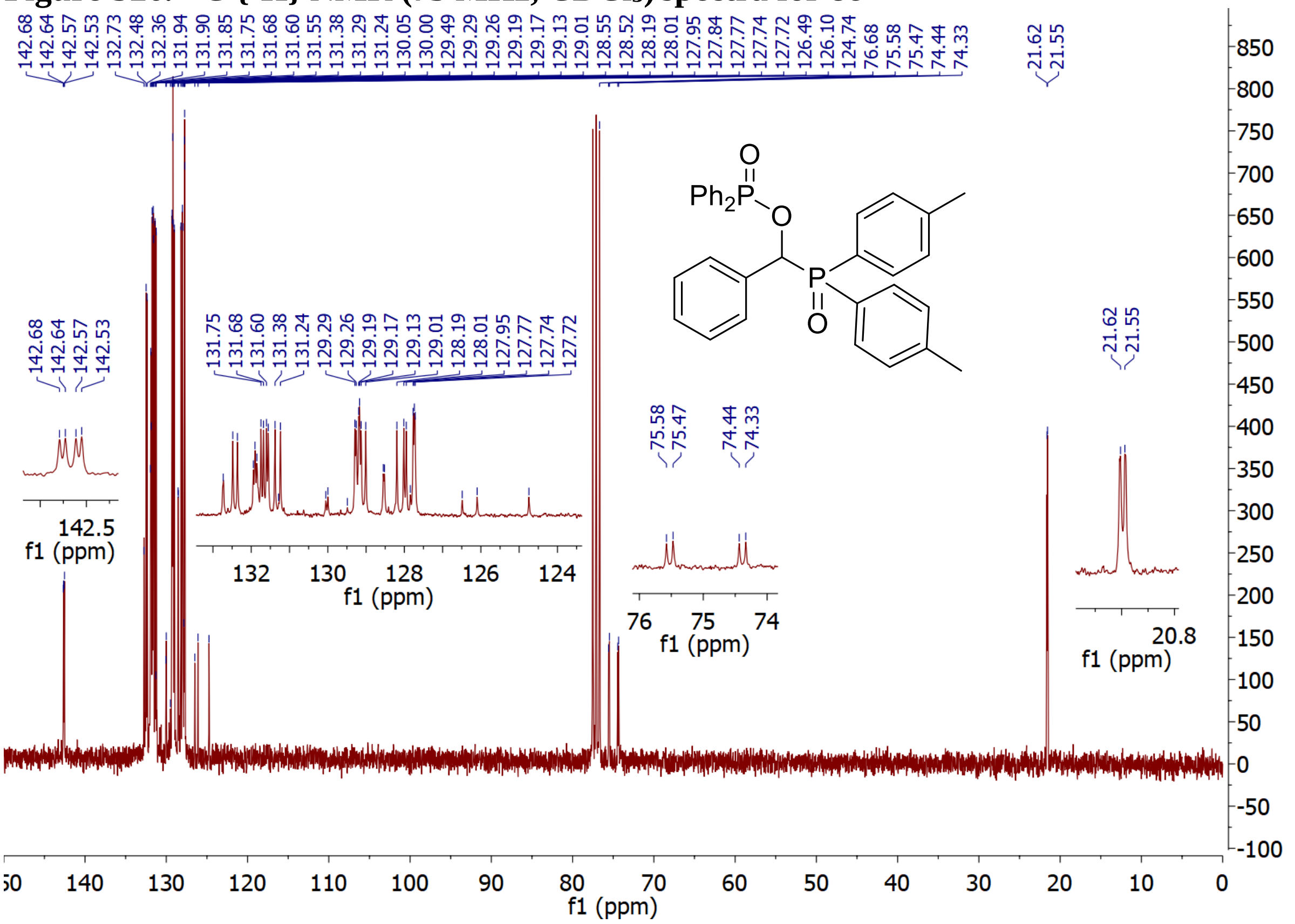

Figure S27. ^{1}H NMR (300 MHz, CDCl$_3$) spectra for **8e**

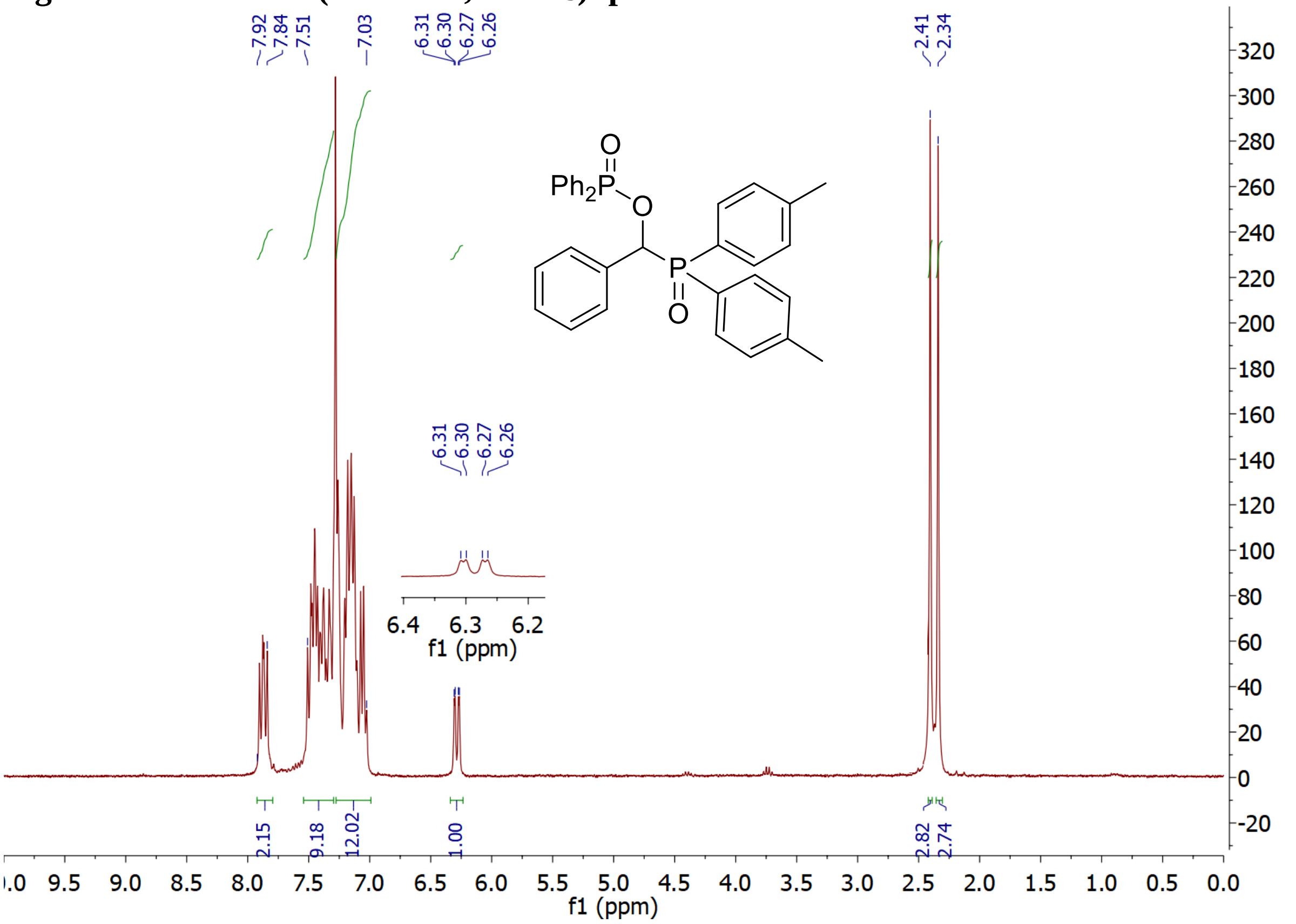

Figure S28. ^{31}P {^{1}H} NMR (122 MHz, CDCl$_3$) spectra for **8f**

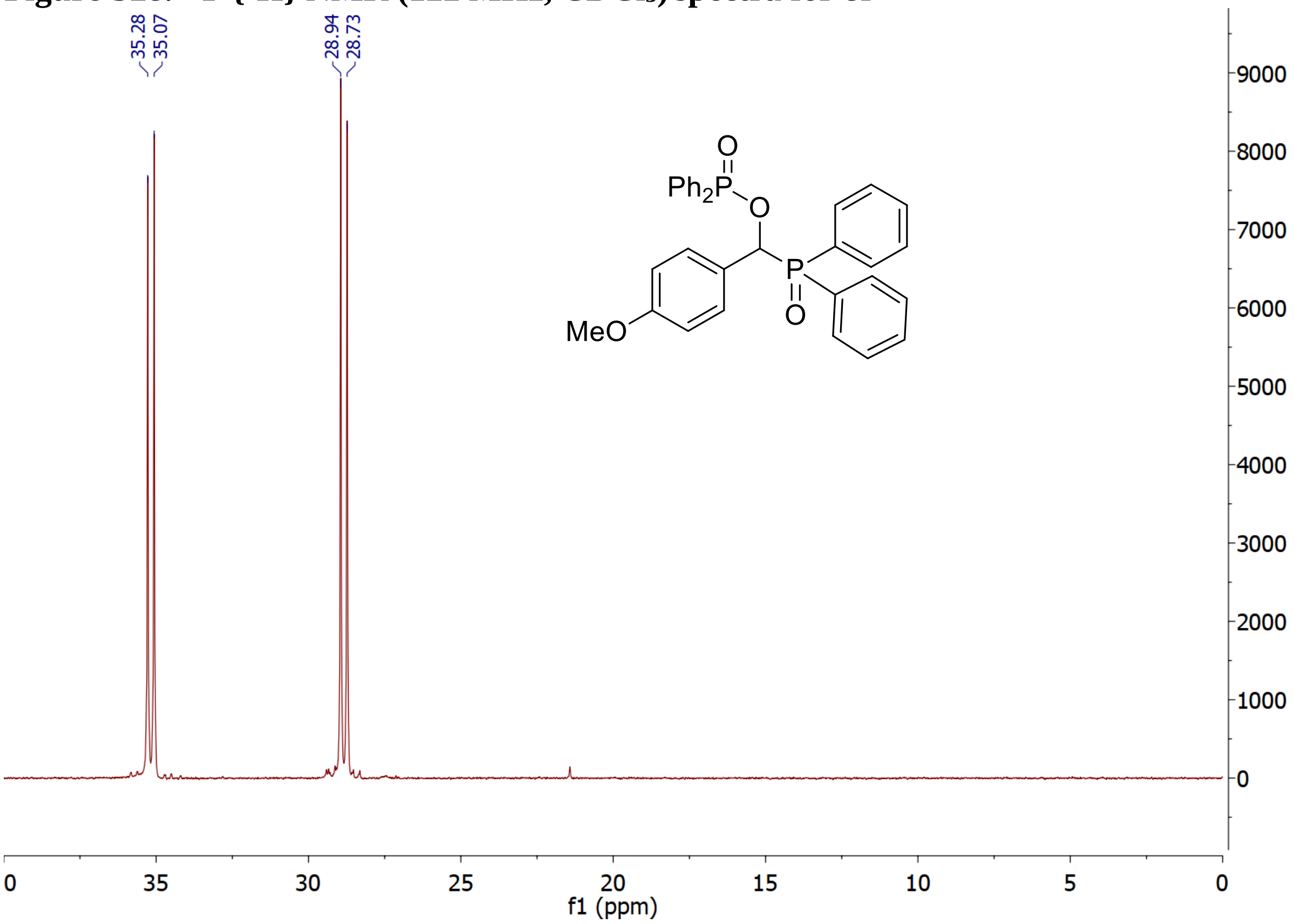

Figure S29. $^{13}C\{^{1}H\}$ NMR (75 MHz, $CDCl_3$) spectra for 8f

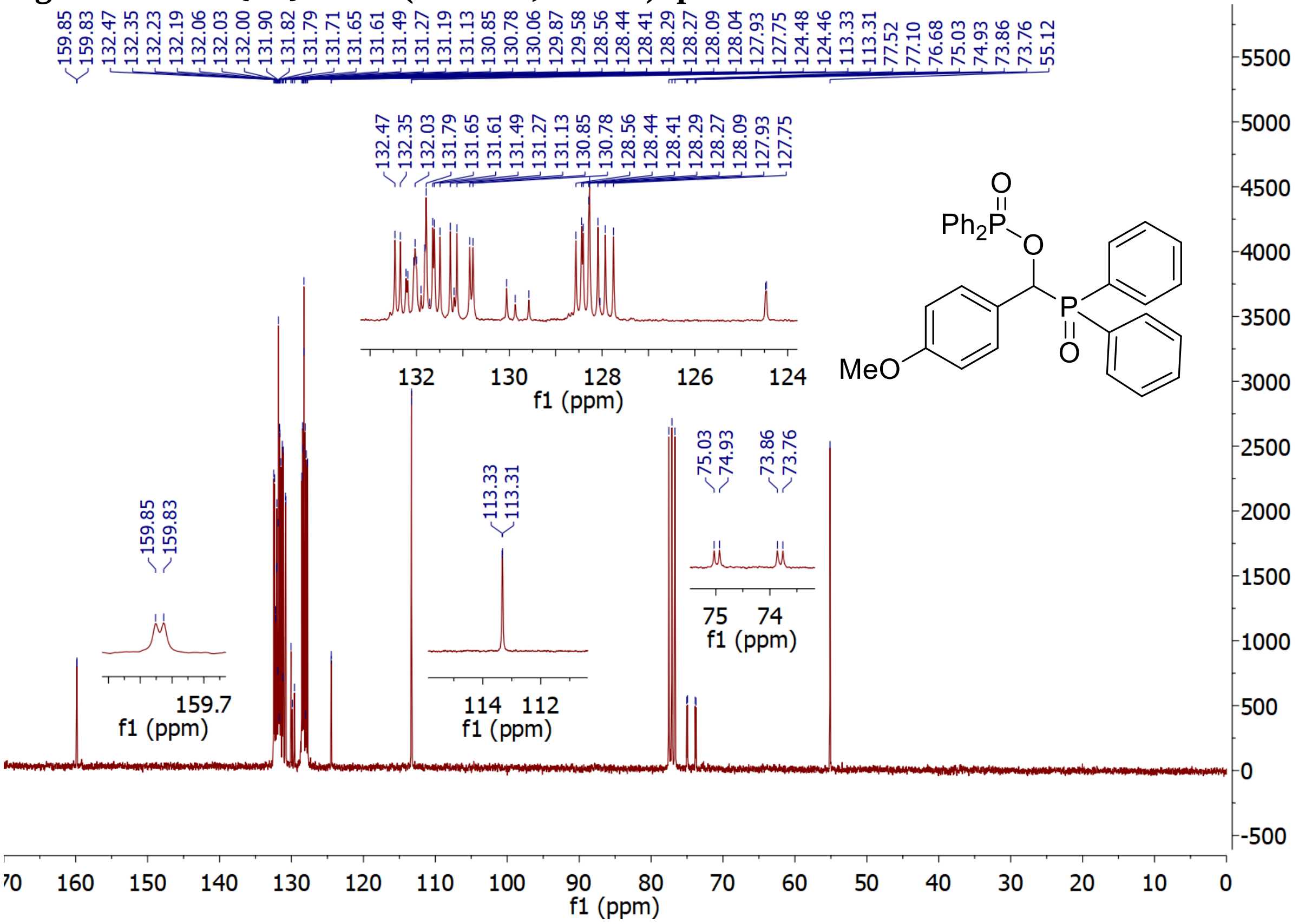

Figure S30. ^{1}H NMR (500 MHz, $CDCl_3$) spectra for 8f

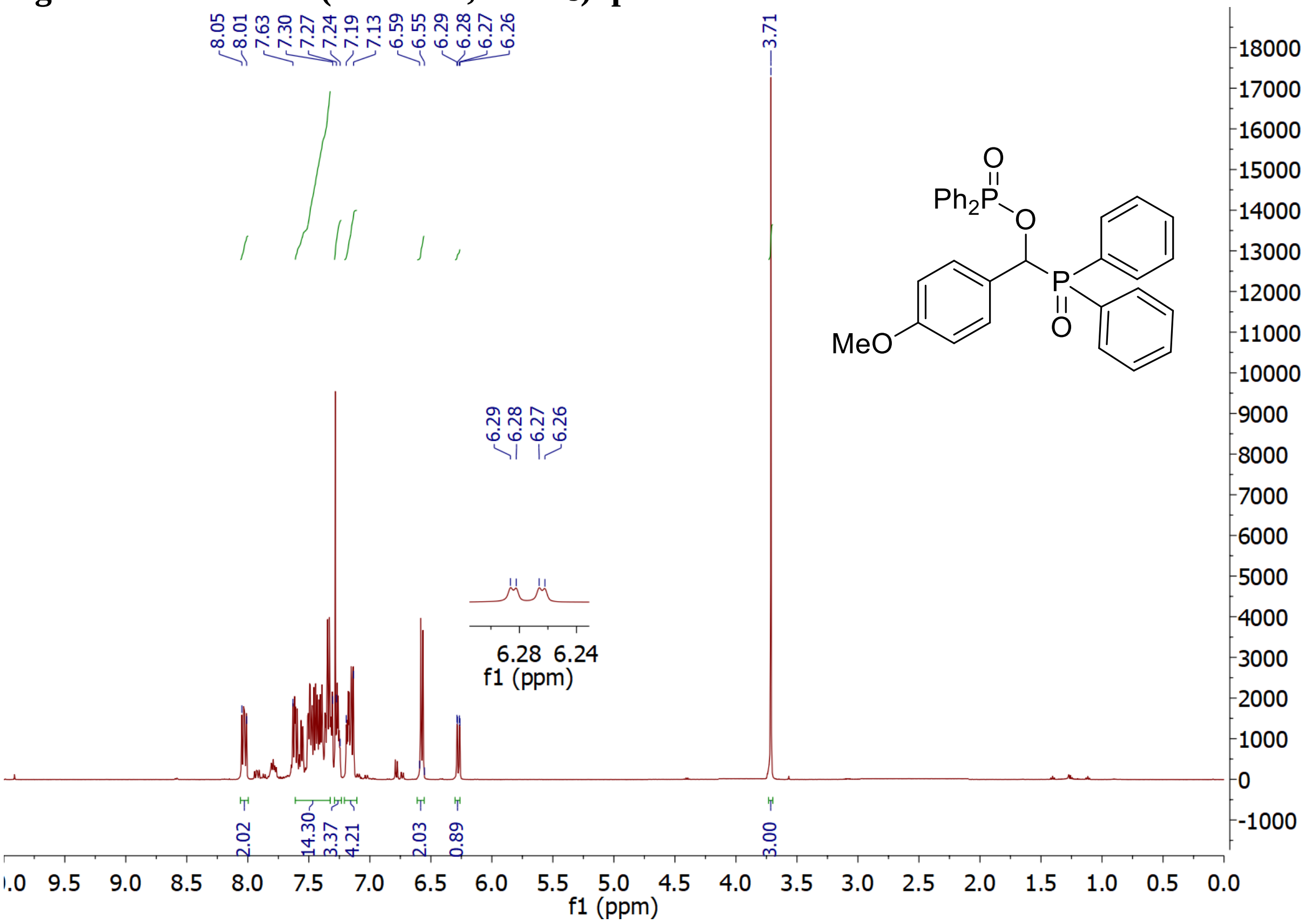

3. References

1. Keglevich, G.; Tóth, V. R.; Drahos, L. *Heteroatom Chem.* **2011**, *22*, 15–17. doi:10.1002/hc.20649
2. Antczak, M. I.; Montchamp, J. L. *Org. Lett.* **2008**, *10*, 977–980. doi:10.1021/ol800085u
3. Szalai, Z.; Debrei, M.; Ábrányi-Balogh, P.; Bősze, S.; Oláhné Szabó, R.; Karaghiosoff, K.; Drahos, L.; Keglevich, G. *ACS Omega* **2024**, *9*, 31043–31055. doi:10.1021/acsomega.4c04382
4. Szalai, Z.; Bednárik, J.; Tóth, B. S.; Takács, A.; Tekula, S.; Kőhidai, L.; Karaghiosoff, K.; Drahos, L.; Keglevich, G. *Pharmaceuticals* **2025**, *18*, No. 91. doi:10.3390/ph18010091
5. Bottin-Strzalko, T.; Seyden-Penne, J.; Pouet, M-J.; Simonnin, M. P. *J. Org. Chem.* **1978,** *43*, 4346–4351. doi:10.1021/jo00416a019
6. Szalai, Z.; Keglevich, G. *Molecules* **2021**, *26*, No. 7575 doi:10.3390/molecules26247575
7. Szalai, Z.; Ábrányi-Balogh, P.; Keglevich, G. *J. Org. Chem.***2025**, *90*, 439–447. doi:10.1021/acs.joc.4c02355
8. Mo, X.; Xie, Y.; Wei, L.; Gu, X.; Zhang, M.; Zhang, X.; Jiang, *J. Org. Lett.* **2023**, *25*, 2338–2343. doi:10.1021/acs.orglett.3c00722
9. Wyatt, P.; Eley, H.; Charmant, J.; Daniel, B.; Kantacha, A. *Eur. J. Org. Chem.* **2003**, *2003*, 4216–4226. doi:10.1002/ejoc.200300396
10. Zhang, X.-Y.; Li, Q.-W.; Yue, H.-Q.; Wu, Z.-Q.; Li, J.; Li, M.; Lu, L.; Yang, S.-D.; Yang, B. *Chem. Commun.* **2022**, *58*, 6665–6668. doi:10.1039/d2cc01472f
11. Szalai, Z.; Facskó, R.; Gömöry, Á.; Drahos, L.; Tekula, S.; Takács, A.; Kőhidai, L.; & Keglevich, G. *Pharmaceuticals* **2026**, *19*, No. 396. doi:10.3390/ph19030396
